# Supplementary material for: Survey of the effect of viewing an online e-cigarette advertisement on attitudes towards cigarette and e-cigarette use in adults located in the UK and USA: a cross-sectional study
Source: BMJ Open. 2019 Jun 18;9(6):e027525. doi: 10.1136/bmjopen-2018-027525 (PMC6589002; doi:10.1136/bmjopen-2018-027525)
Supplement: Author's manuscript [file bmjopen-2018-027525.draft_revisions.pdf]

BMJ Open is committed to open peer review. As part of this commitment we make the peer review history of every article we publish publicly available.

When an article is published we post the peer reviewers' comments and the authors' responses online. We also post the versions of the paper that were used during peer review. These are the versions that the peer review comments apply to.

The versions of the paper that follow are the versions that were submitted during the peer review process. They are not the versions of record or the final published versions. They should not be cited or distributed as the published version of this manuscript.

BMJ Open is an open access journal and the full, final, typeset and author-corrected version of record of the manuscript is available on our site with no access controls, subscription charges or pay-per-view fees (http://bmjopen.bmj.com).

If you have any questions on BMJ Open's open peer review process please email info.bmjopen@bmj.com

BMJ Open

The effect of viewing an on-line e-cigarette advertisement on attitudes towards tobacco smoking and e-cigarette use: an experimental study.

Journal:	*BMJ Open*
Manuscript ID	bmjopen-2018-027525
Article Type:	Research
Date Submitted by the Author:	29-Oct-2018

Complete List of Authors:	Booth, Paula; University of East London, Psychology<br>Albery, Ian; London South Bank University Department of Applied Sciences, Division of Psychology (School of Applied Sciences)<br>Cox, Sharon; London South Bank University Department of Applied Sciences, Division of Psychology (School of Applied Sciences)<br>Frings, Daniel; London South Bank University Department of Applied Sciences, Division of Psychology (School of Applied Sciences)

Keywords:	attitude, advertisement, e-cigarettes, tobacco, dual-users, smokers

SCHOLARONE™
Manuscripts

The effect of viewing an on-line e-cigarette advertisement on attitudes towards tobacco smoking and e-cigarette use: an experimental study

Paula Booth[1], Ian. P. Albery[2], Sharon Cox[2] & Daniel Frings[2]

[1](Paula Booth – corresponding author) Applied Health and Communities College, School of Psychology, Psychological Sciences Department, The University of East London, Stratford Campus, Water Lane

London, E15 4LZ. p.booth@uel.ac.uk

Email: p.booth@uel.ac.uk

Phone: +44 (0)20 8223 4719

[2]Division of Psychology, School of Applied Sciences, London South Bank University, 103 Borough Road, London, SE1 0AA

Key words: E-Cigarette; Tobacco; Attitudes; Advertisement; Dual-Users

Word Count : 2901

For peer review only

Objective

This study tested whether exposure to an on-line electronic cigarette advertisement changed attitudes towards tobacco smoking and e-cigarette in smokers, non-smokers, e-cigarette users and dual-users (smokers who also use an e-cigarette).

Methods

Adult participants (n=964) from the UK and USA participated in an on-line survey. Amongst different participant groups (non-smokers, e-cigarette users, dual-users and smokers), perceptions of healthiness, desirability and social acceptability of tobacco and e-cigarettes were measured before and immediately after viewing an on-line e-cigarette advertisement.

Results

Post exposure all groups showed a decrease or no change in how socially acceptable or desirable they rated tobacco cigarettes. Paradoxically, dual-users rated tobacco cigarettes as being healthier after viewing the advertisement whilst all other smoking group ratings remained the same. There was an increase or no change in how all smoking groups perceived the healthiness and desirability of e-cigarettes.

Conclusion

We observed no evidence that exposure to an e-cigarette advertisement renormalizes or encourages tobacco smoking in smokers, non-smokers or e-cigarette users. However, there is some indication that viewing an e-cigarette advertisement may increase duals-users’ perceptions of the health of tobacco smoking. E-cigarette advertising may also support smokers in

For peer review only

transitioning to e-cigarettes, but the effects of advertisements on nicotine naïve groups needs careful monitoring.

Strengths and limitations of this study

- This study is among the first to examine the attitudinal processes underlying the effect of viewing an advertisement on interest in both smoking and vaping.
- The sample were derived of smokers, non-smokers, vapers and dual users rather than focusing on only one smoking type which is often a limitation of the extant literature in this area of research.
- The effects of viewing an advertisement on actual smoking behaviour was not assessed.

For peer review only

INTRODUCTION

There is a large body of evidence to support the notion that advertising tobacco cigarettes encourages people to start or to continue to smoke.[1,2] With the intent of reducing tobacco use globally, Article 13 of the WHO Framework Convention for Tobacco Control calls for "a comprehensive ban on advertising, promotion and sponsorship of tobacco cigarettes"[3] (pg. 11). However, this treaty does not cover e-cigarettes. While there are restrictions on advertising e-cigarettes in the UK (Committee on Advertising Practice)[4] and in the USA (U.S. Food and Drug Regulations)[5] e-cigarettes are still being marketed elsewhere and advertisements can include celebrity endorsements and portray e-cigarettes as being sexy and fun.[6] In these territories the public now experiences increased exposure to e-cigarette advertisements[4,7,8] in the form of television, magazines, newspapers, billboards and internet advertisements.[9] The current paper explores the potential of such advertisements to (i) renormalize smoking and (ii) reduce barriers to e-cigarette uptake amongst current smokers.

One concern related to e-cigarette advertisements is that they may renormalize smoking. The de-normalisation of smoking has been a cornerstone of tobacco control policies in encouraging existing smokers to quit and also to discourage initial uptake[10–12] and social acceptance of smoking has been shown to be a barrier to reduction in smoking prevalence in a variety of communities.[13,14] It is therefore important to determine whether e-cigarette advertising may also affect attitudes towards tobacco cigarettes by making them more positive. A number of researchers have suggested that e-cigarette advertising could lead to increases in how socially acceptable and desirable tobacco cigarettes are perceived to be, and subsequently influence their continued use or possible (re)uptake in smokers, e-cigarette users and dual-users (those who use

For peer review only

both tobacco and e-cigarette products).[6,15–17]. Alongside observed effects on these groups, concerns that e-cigarette use may be a gateway to tobacco smoking suggests it is also important to determine whether advertising may encourage non-smokers to use e-cigarettes.[16,17] In this context, it is important to note that the evidence base around actual usage statistics currently suggests that e-cigarette availability does not appear to have led to uptake of either e-cigarettes or tobacco products amongst British non-smokers: the current number of UK non-smokers using an e-cigarette daily is less than 1%, and tobacco use rates continue to decline.[18] A similar pattern is observed in the US.[19] However, results from laboratory studies exploring the effects of advertisements on urges to smoke and interest in smoking amongst other groups are inconsistent, and none have examined attitudinal processes which may underlie those effects which have been observed.[20–22] The current study aims to examine the issue of renormalisation by measuring attitude change towards tobacco cigarettes in adult non-smokers, smokers, e-cigarette users and dual-users before and after viewing an advertisement.

In contrast to concerns about renormalisation, e-cigarette advertisements may represent an opportunity to promote reduced risk products as an alternative to smoking. Although there are currently only relatively few studies comparing the health effects of e-cigarettes use to smoking or non-smoking over time,[23,24] in the UK e-cigarettes are widely supported as a reduced risk nicotine alternative for smokers, and has received support from a number of agencies. [18,25–27] However in the UK, despite increasing public agency support, one barrier to the uptake of e-cigarettes amongst smokers has been an increasingly negative attitudes towards the products. Between 2015 and 2017 the proportion of UK smokers who believed that e-cigarettes are less harmful than tobacco cigarettes reduced from 31% to 20% (Action on Smoking and Health)[27]. Similarly, in the USA the percentage of smokers who perceived e-cigarettes to be equally

For peer review only

harmful than combustible cigarettes increased from 23% in 2012 to 35% in 2015.[28] To the extent attitude predict behaviour, such negative perceptions of the health of e-cigarettes are likely to reduce the potential for e-cigarettes to be used as a smoking cessation aid. E-cigarette advertising may serve to halt or reverse this decline: Research examining the effects of advertising on perception of e-cigarettes (carried out predominantly on younger populations) shows that viewing an advertisement may increase the perception of social acceptability and the intention to try an e-cigarette in the future.[7,29] For instance, 13-17 year old smokers showed a more positive attitude towards e-cigarettes after viewing an e-cigarette advertisement,[29] and adult smokers reported a decrease in the acute urge to smoke[30] and an interest in trying e-cigarettes.[31–33] Thus, in the current study we also explore the potential for e-cigarette advertisements to change the way smokers (and other groups) perceive the healthiness, socially acceptability and general desirability of e-cigarettes.[13,34]

One limitation of the extant literature in these areas is that it focuses on either tobacco users, non-smokers and/or e-cigarette users. This leaves one important population, dual-users, understudied. In a survey of e-cigarette use in Europe, of an estimated 37 million e-cigarette users, 73% were currently still smoking.[35] Many dual-users use e-cigarettes in areas in which they are unable to smoke tobacco cigarettes.[36] However, they still have a preference for smoking tobacco cigarettes in stressful situations or for pleasure.[37] Thus, it is important to establish whether viewing an e-cigarette advertisement can change attitudes towards smoking in dual-users.

To explore the potential for-cigarette advertisements to (i) renormalize tobacco and/or (ii) reduce barriers to e-cigarettes uptake, the current study investigated whether viewing an e-cigarette advertisement influenced the extent to which tobacco and e-cigarettes were perceived to be more

For peer review only

or less healthy, desirable and socially acceptable in dual-users, smokers, non-smokers and e-cigarette users.

METHODS

Design

A cross sectional design in which attitude measures were taken pre and post advertisement viewing. The between-subjects factor was smoking group (non-smokers, e-cigarette users, dual-users and smokers). The dependent variables were the scores on a series of questions rating attitudes around the healthiness, desirability and social acceptability of both e-cigarettes and tobacco cigarettes.

Participants, Recruitment and Procedure

Originally, 964 participants completed the survey, between December 2015 and February 2016, but data from 199 were removed for being under the age of 18 or not entering their age, failing controls (see below) and having missing data. Data were included for 765 participants, 361 male, 400 female and 4 others from an age range of 18-65 years old (M = 36 years, SD = 11.6). Participants were defined as smokers (n=115), non-smokers (410), e-cigarette users (100) or dual-users (145). They were located in the USA (n=543) or the UK (n=222).

Participants were recruited through an online crowd-sourcing tool (Crowdflower, similar to MTurk) on which a link to a survey (delivered using Qualtrics) was placed or Twitter. Participants were paid a fee of $1.00 to complete the survey. Control questions were built into the Qualtrics survey to exclude automated responses and participants who were not concentrating appropriately on the questions (for example, “For this item please indicate strongly agree”). Additional controls were set up on the survey so that participants were unable to enter the site

For peer review only

from the same IP address more than once and that they had to take a minimum time of 90 seconds to complete the survey. Post-hoc analysis indicated that a minimum of 100 participants (the smallest smoking group) was sufficient to detect an effect size r =.15 or greater, (with Z = 2.2 or above).

Patient and public involvement.

Patients were not involved in the development or conduct of this study. There are plans to disseminate the results to practitioners by including the results as an aspect in London South Bank courses provision aimed at service delivery managers and counsellors. A summary of the findings will be offered to Cancer Research UK and other policy organisations and promoted on relevant staff social media sites.

Measures

Attitudes towards Cigarettes

Participants rated how *healthy*, how *desirable* and how *socially acceptable* they found tobacco and e- cigarettes on a 7-point Likert type scale ranging from 1 '*strongly disagree'* to 7 '*strongly agree*.' They were given two separate statements: "When thinking about tobacco/e- cigarettes please indicate whether you think they are……." The order in which e-cigarettes and tobacco cigarette statements were presented were counter-balanced to reduce question order bias.

Smoking Status

Participants were asked to indicate how often (never, occasionally, very often, always) they smoked, vaped or used nicotine products both in the present and the past. Participants were placed in smoking groups based on their 'present' smoking status. Those that answered 'never'

For peer review only

to using tobacco or e-cigarettes were defined as non-smokers, those that answered ‘occasionally’, ‘very often’ or ‘always’ were defined as smokers, e-cigarette users or dual-users as appropriate.

Procedure

The study was given ethical approval by the local University Research Ethics Committee. Participants were asked to rate how healthy, desirable and socially acceptable they found tobacco and e-cigarettes before and after viewing one of fifteen e-cigarette advertisements (see below). Next, they were asked to rate the emotional attributes and the perceived effectiveness of the advertisement. They were then asked for demographic characteristics. Finally, they were given a debrief about the study, a warning about the addictive nature of nicotine and given links to government quit smoking websites.

Advertisement Selection and Preparation

An account of advertisement selection and preparation is reported in the protocol of a larger study which investigated the effects of viewing an e-cigarette advertisement on explicit and implicit attitudes (see Booth, Albery & Frings, 2017). The current study and the larger study were funded by Cancer Research UK (CRUK grant number C54622/A20485). The data reported here were used to identify which advertisement would be used in this larger study.

RESULTS

Health scores in the smokers group were not normally distributed due to the positive skew (baseline skewness = 2.63, test skewness = 2.40) thus Wilcoxon Signed Rank Tests were used to compare pre and post intervention scores in each smoking group (smokers, non-smokers, dual-users and e-cigarette users) (see Table 1).

For peer review only

Table 1. Mean healthy, desirability and social acceptability ratings (standard deviation in parentheses) towards tobacco and e-cigarettes by smoking group pre and post advertisement presentation.

		Sample							
Attitude target	*Attitude dimension*	Non-smoker (n=410)		E-cigarette user (n=100)		Smoker (n=115)		Dual-User (n= 145)	
		Pre	Post	Pre	Post	Pre	Post	Pre	Post
Tobacco Cigarette	Healthy	1.27 (0.83)	1.28 (0.76)	1.47 (0.90)	1.47 (0.81)	1.59 (0.90)	1.73 (1.03)	2.08 (1.35)	**2.26**** **(1.50)**
	Desirability	1.86 (1.45)	1.77 (1.35)	2.84 (1.79)	**2.58*** **(1.67)**	3.52 (1.78)	**3.21**** **(1.67)**	3.85 (1.87)	3.71 (1.82)
	Social Acceptability	2.63 (1.57)	**2.39***** **(1.54)**	3.04 (1.76)	**2.86*** **(1.78)**	3.43 (1.55)	3.30 (1.64)	3.36 (1.67)	3.39 (1.77)
E-Cigarette	Healthy	2.53 (1.38)	**2.68**** **(1.51)**	4.79 (1.60)	4.82(1.65)	3.03 (1.48)	**3.21***** **(1.54))**	3.80 (1.33)	**4.01**** **(1.42)**
	Desirability	2.76 (1.57)	**2.91*** **(1.70)**	5.36 (1.44)	5.21 (1.37)	3.91 (1.45)	3.91 (1.54)	4.82 (1.21)	**5.03**** **(1.24)**
	Social Acceptability	3.93 (1.58)	**3.81*** **(1.07)**	5.08 (1.45)	5.07 (1.46)	4.59 (1.39)	4.50 (1.40)	5.28 (1.05)	5.14 (1.18)

Note: Comparisons are made pre-post for each dimension for each group, with significant differences marked with asterisk(es): * = $p<0.05$ **= $p<0.005$ ***= $p<0.001$

Attitudes Towards Tobacco Cigarettes

All smoking groups gave low scores for health of tobacco cigarettes at baseline. Showing an e-cigarette advertisement made no significant difference to the score in the smoking, non-smoking and e-cigarette user groups but dual-users scored tobacco cigarettes as healthier after viewing the advertisement ($Z = 2.57$ $p = .01$). At baseline, dual-users had higher scores for health, desirability and social acceptability than any other smoking group but this group showed no changes in desirability or social acceptability. E-cigarette users scored tobacco cigarettes as significantly less desirable ($Z = -2.50$, $p = .013$) and less socially acceptable ($Z = -2.501$ $p = .012$) after viewing an advertisement. Smokers scored tobacco cigarettes as less desirable ($Z = -2.81$, $p = .005$) after viewing an advertisement but there was no difference in pre and post scores of social acceptability. Non-smokers showed no change in desirability scores of tobacco cigarettes but scored them as less socially acceptable ($Z = -4.67$ $p > .001$) after viewing an advertisement.

Attitudes Towards E-Cigarettes

All groups scored e-cigarettes as healthier, desirable and socially acceptable than tobacco cigarettes at baseline. Non-smokers scored e-cigarettes as being healthier after viewing an e-cigarette advertisement ($Z = 2.97$, $p = .003$), more desirable ($Z = 2.60$, $p = .009$) but less socially acceptable ($Z = -2.12$ $p = .034$). E-cigarette users showed no change in attitudes. Smokers scored e-cigarettes as healthier after viewing the advertisements ($Z = 2.21$, $p = .027$) but there was no change in desirability or social acceptability. Dual-users scored e-cigarettes as being healthier after viewing advertisement ($Z = 2.53$, $p = .011$) and more desirable ($Z = 2.04$, $p = .042$) but there was no change in social acceptability.

DISCUSSION

For peer review only

The current study aimed to explore the potential of e-cigarette advertising to (i) renormalize tobacco products by reducing negative evaluations towards them and (ii) reduce barriers to the uptake of e-cigarettes by smokers. The results of the study showed that before viewing the e-cigarette advertisement all groups found e-cigarettes to be more desirable, socially acceptable and healthier than cigarettes. These results are reassuring in view of the findings that a significant proportion of the public perceive e-cigarettes to be as, or more, harmful as tobacco cigarettes, in line with emerging evidence base.[27,28]

In terms of renormalisation, little evidence in the current study supports the notion that viewing e-cigarette adverts increased positive evaluations of tobacco cigarettes. Rather, perceptions of tobacco cigarettes being socially acceptable and desirable generally decreased. In non-smokers, smokers and e-cigarette users there was no change in health scores for cigarettes. However, these smoking groups gave scores close to the scale's floor for health at baseline and thus there may have been no possibility to reduce scores further after viewing the advertisement. Thus, there was no indication that viewing an e-cigarette advertisement renormalized or encouraged smoking amongst these groups.

In terms of reducing barriers to uptake, viewing an advertisement increased the perception that e-cigarettes are healthy amongst smokers, dual-users and non-smokers. These results suggest that e-cigarette advertising may encourage smokers to perceive e-cigarettes more positively. As perceptions of health are a key determinant in behaviour change these results encourage the view that smokers may potentially use e-cigarettes as a tool for smoking cessation.[38] Non-smokers perceived tobacco and e-cigarettes to be less healthy, socially acceptable and desirable than any of the other smoking groups. After viewing an advertisement, the scores for social acceptability of tobacco and e-cigarettes decreased further. This result is inconsistent with previous findings

which found that when young people viewed e-cigarette advertisements the ratings for social acceptability increased.[7,29] This discrepancy in results may be due to the different age groups being tested and the suggestion that e-cigarette adverts (and products) are often designed with a younger target audience in mind.[39] It also mirrors a more general negative attitude towards e-cigarettes observed in the UK population in recent years.[27] However, in the current study, after viewing the advertisement the perception that e-cigarettes are healthy and desirable increased amongst non-smokers. Although widespread use of e-cigarettes by non-smokers has not been observed to date (in populations where data is available), this result suggests the effects of e-cigarettes advertising on actual e-cigarette uptake amongst nicotine naïve users should continue to be monitored, especially in territories where advertising is prevalent.

Of all the smoking groups e-cigarette users scored e-cigarettes as the healthiest and desirable before viewing the advertisement and showed no change in attitude after viewing. Perceptions of desirability and social acceptability of tobacco cigarettes decreased after watching the advertisement. Thus, viewing an e-cigarette advertisement may help deter ex-smokers, who are vaping as a smoking cessation tool, from relapsing.

One group who are relatively under-researched (dual-users) displayed a different pattern of responses to tobacco product to other smokers. They had the highest scores for health, desirability and social acceptability of tobacco cigarettes pre and post viewing of the e-cigarette advertisement compared to any other smoking group. These results are consistent with another study in which dual-users rated tobacco cigarette smoking as more desirable and healthier than any other smoking group (Frings, The Effect of Anti-Smoking Messages on Attitudes Towards Electronic-Cigarettes). After viewing the advertisement they perceived both tobacco and e-cigarettes as healthier and e-cigarettes as more desirable than before viewing. The contradictory

For peer review only

effect of the advertisements on the attitudes of this particular group suggests that the drive to use both tobacco and e-cigarettes is complex. Indeed, research suggests that many dual-users may still use tobacco cigarettes because they find it to be a pleasurable experience and use e-cigarettes as a practical solution when smoking tobacco cigarettes is banned.[37] Further research is required to identify the best strategy to help motivate this group to quit smoking.

Study Limitations

One of the limitations of this study was that we only measured attitudes towards tobacco and e-cigarettes but did not assess any effects on intention to smoke or actual smoking behaviour. Furthermore, the change in attitude was measured immediately after one exposure to the advertisement so it cannot be determined whether effects of viewing an advertisement would persist over a longer time period. Future research would benefit from assessing changes in attitude longitudinally as well as measuring intentions to smoke or use e-cigarettes and actual smoking behaviour for a fixed period before and after viewing the advertisement.

The measure of attitude could also be improved in future research. In particular, measuring attitudes towards health using a seven-point Likert type scale resulted in possible floor effects. A more sensitive measure such as a Visual Analogue Scale may have given a broader range of data ensuring that the data was more normally distributed and giving a better indication of changes that may have occurred at the lower end of the scale. In addition, the attitudes were assessed using explicit, self-report measures. There is a social stigma associated with smoking and so there may have been an effect of social desirability bias. Thus, an implicit measure of attitude could be measured in future research as previous research shows that these measures of attitude interact to predict actual smoking behaviour.[40]

For peer review only

Conclusion

The results from this study suggest that viewing an e-cigarette advertisement is unlikely to renormalize tobacco smoking amongst most groups. However, we also observed some evidence that e-cigarette adverts may increase how healthy tobacco products are perceived amongst dual-users. This highlights the importance of more research with this relatively understudied group, in particular around factors which promote the decision to transition from dual-use to e-cigarette use or nicotine abstinence. In terms of reducing barriers to uptake, e-cigarette advertisement may encourage them to quit using e-cigarettes as a smoking cessation tool. Thus, e-cigarette advertisements could be considered a viable tool to stimulate or support smoking cessation. However, where e-cigarette advertising is prevalent, careful monitoring of uptake of e-cigarettes amongst nicotine naïve populations is important.

For peer review only

References

1 Evans N, Farkas A, Gilpin E, *et al.* Influence of Tobacco Marketing and Exposure to Smokers on Adolescent Susceptibility to Smoking. *JNCI J Natl Cancer Inst* 1995;**87**:1538–45.
2 Tye JB, Warner KE, Glantz SA. Tobacco Advertising and Consumption: Evidence of a Causal Relationship. *J Public Health Policy* 1987;**8**:492.
3 WHO FRAMEWORK CONVENTION ON TOBACCO CONTROL WHO Library Cataloguing-in-Publication Data. 2010. http://apps.who.int/iris
4 New rules for the marketing of e-cigarettes CAP and BCAP's Joint Regulatory Statement. https://www.asa.org.uk/asset/851604F6-C081-420C-9DDDA65124B775A4/ (accessed 21 Aug 2017).
5 Commissioner O of the. Economic Impact Analyses of FDA Regulations - Summary: Deeming Tobacco Products to be Subject to the Food, Drug, and Cosmetic Act, as Amended by the Family Smoking Prevention and Tobacco Control Act; Regulations Restricting the Sale and Distribution of Tobacco Products and Required Warning Statements for Tobacco Product Packages and Advertisements (Final Rule). https://www.fda.gov/AboutFDA/ReportsManualsForms/Reports/EconomicAnalyses/ucm500249.htm (accessed 16 Apr 2018).
6 De Andrade M, Hastings G, Angus K, *et al.* the marketing of electronic cigarettes in the uk. 2013. https://dspace.stir.ac.uk/bitstream /1893/17889/1/deAndradeetale-cigsreport.pdf (accessed 26 Mar 2017).
7 Duke JC, Lee YO, Kim AE, *et al.* Exposure to Electronic Cigarette Television Advertisements Among Youth and Young Adults. *Pediatrics* 2014;**134**.
8 Basch CH, Mongiovi J, Hillyer GC, *et al.* An Analysis of Electronic Cigarette and Cigarette Advertising in US Women's Magazines. *Int J Prev Med* 2016;**7**:103.
9 Bauld L, Angus K, De Andrade M, *et al.* Electronic Cigarette Marketing: Current Research and Policy. 2016. http://www.cancerresearchuk.org/sites/default/files/ electronic_cigarette_marketing_report_final.pdf (accessed 21 Aug 2017).
10 Linkenbach, J. W., & Perkins HW. MOST of us are tobacco free: An eight-month social norms campaign reducing youth initiation of smoking in Montana. In: H. W. Perkins, ed. *The social norms approach to preventing school and college age substance abuse: A handbook for educators, counselors, and clinicians*. San Francisco, CA, US: : Jossey-Bass 2003. 224–34.
11 Reid JL, Manske SR, Leatherdale ST. Factors related to adolescents' estimation of peer smoking prevalence. *Health Educ Res* 2007;**23**:81–93.
12 Bayer R, Bachynski KE. Banning Smoking In Parks And On Beaches: Science, Policy, And The Politics Of Denormalization. *Health Aff* 2013;**32**:1291–8.
13 Nicholson AK, Borland R, van der Sterren AE, *et al.* Social acceptability and desirability of smoking in a national sample of Aboriginal and Torres Strait Islander people. *Med J Aust* 2015;**202**:57–62.
14 Twyman L, Bonevski B, Paul C, *et al.* Perceived barriers to smoking cessation in selected vulnerable groups: a systematic review of the qualitative and quantitative literature. *BMJ Open* 2014;**4**:e006414.
15 Fairchild AL, Bayer R, Colgrove J. The Renormalization of Smoking? E-Cigarettes and the Tobacco "Endgame". *N Engl J Med* 2014;**370**:293–5.

For peer review only

16 Schneider S, Diehl K. Vaping as a Catalyst for Smoking? An Initial Model on the Initiation of Electronic Cigarette Use and the Transition to Tobacco Smoking Among Adolescents. *Nicotine Tob Res* 2016;**18**:647–53.
17 Etter J-F. Gateway effects and electronic cigarettes. *Addiction* Published Online First: 7 August 2017. doi:10.1111/add.13924
18 Mcneill A, Brose LS, Calder R, *et al.* Evidence review of e-cigarettes and heated tobacco products 2018 A report commissioned by Public Health England. 2018.
19 *QuickStats:* Cigarette Smoking Status* Among Current Adult E-cigarette Users,† by Age Group — National Health Interview Survey,§ United States, 2015. *MMWR Morb Mortal Wkly Rep* 2016;**65**:1177. https://www.cdc.gov/mmwr/volumes/65/wr/mm6542a7.htm (accessed 12 Sept, 2018)
20 Maloney EK, Cappella JN. Does Vaping in E-Cigarette Advertisements Affect Tobacco Smoking Urge, Intentions, and Perceptions in Daily, Intermittent, and Former Smokers? *Health Commun* 2015;:1–10.
21 Durkin SJ, Bayly M, Wakefield MA. Can E-cigarette Ads Undermine Former Smokers? An Experimental Study. *Tob Regul Sci* 2016;**2**:263–77.
22 Smith DM, Bansal-Travers M, O'Connor RJ, *et al.* Associations between perceptions of e-cigarette advertising and interest in product trial amongst US adult smokers and non-smokers: results from an internet-based pilot survey. *Tob Induc Dis* 2015;**13**:14.
23 Shahab L, Goniewicz ML, Blount BC, *et al.* Nicotine, Carcinogen, and Toxin Exposure in Long-Term E-Cigarette and Nicotine Replacement Therapy Users. *Ann Intern Med* 2017;**166**:390.
24 Polosa R, Cibella F, Caponnetto P, *et al.* Health impact of E-cigarettes: a prospective 3.5-year study of regular daily users who have never smoked. *Sci Rep* 2017;**7**:13825.
25 E-cigarettes | Cancer Research UK. http://www.cancerresearchuk.org/about-cancer/causes-of-cancer/smoking-and-cancer/e-cigarettes (accessed 16 Apr 2018).
26 Royal College of Physicians. Nicotine without smoke: Tobacco harm reduction | RCP London. https://www.rcplondon.ac.uk/projects/outputs/nicotine-without-smoke-tobacco-harm-reduction-0/ (accessed 18 Dec 2017).
27 ASH Action on Smoking and Health. Use of electronic cigarettes (vapourisers) among adults in Great Britain. ASH Fact Sheet 2017. ttp://ash.org.uk/information-and-resources/fact-sheets/use-of-e-cigarettes-among-adults-in-great-britain-2017/ (accessed 8 Aug 2018)
28 Majeed B, Weaver S, Gregory KR, *et al.* Changing perceptions of harm of e-cigarettes among US adults, 2012–2015. *Am J Prev Med* 2017 Mar 1;52(3):331-8.
29 Farrelly MC, Duke JC, Crankshaw EC, *et al.* A Randomized Trial of the Effect of E-cigarette TV Advertisements on Intentions to Use E-cigarettes. *Am J Prev Med* 2015;**49**:686–93.
30 Jo CL, Golden SD, Noar SM, *et al.* Effects of E-cigarette Advertising Messages and Cues on Cessation Outcomes. *Tob Regul Sci* 2018;**4**:562–72.
31 Pepper JK, Brewer NT. Electronic nicotine delivery system (electronic cigarette) awareness, use, reactions and beliefs: a systematic review. *Tob Control* 2014;**23**:375–84.
32 Kim AE, Lee YO, Shafer P, *et al.* Adult smokers' receptivity to a television advert for electronic nicotine delivery systems. *Tob Control* 2015;**24**:132–5.
33 Smith DM, Bansal-Travers M, O'Connor RJ, *et al.* Associations between perceptions of e-cigarette advertising and interest in product trial amongst US adult smokers and non-

For peer review only

smokers: results from an internet-based pilot survey. *Tob Induc Dis* 2015;**13**:14
34 Chapman S, Freeman B. Markers of the denormalisation of smoking and the tobacco industry. *Tob Control* 2008;**17**:25–31.
35 Farsalinos KE, Poulas K, Voudris V, *et al.* Electronic cigarette use in the European Union: analysis of a representative sample of 27 460 Europeans from 28 countries. *Addiction* 2016;**111**:2032–40.
36 Adriaens K, Van Gucht D, Baeyens F. Differences between Dual Users and Switchers Center around Vaping Behavior and Its Experiences Rather than Beliefs and Attitudes. *Int J Environ Res Public Health* 2017;**15**:12.
37 Maglia M, Caponnetto P, Di Piazza J, *et al.* Dual use of electronic cigarettes and classic cigarettes: a systematic review. *Addict Res Theory* 2017;:1–9.
38 Webb TL, Joseph J, Yardley L, *et al.* Using the internet to promote health behavior change: a systematic review and meta-analysis of the impact of theoretical basis, use of behavior change techniques, and mode of delivery on efficacy. *J Med Internet Res* 2010;**12**:e4.
39 Richardson A, Ganz O, Vallone D. Tobacco on the web: surveillance and characterisation of online tobacco and e-cigarette advertising. *Tob Control* 2015;**24**:341–7.
40 Perugini M. Predictive models of implicit and explicit attitudes. *Br J Soc Psychol* 2005;**44**:29–45.

Funding statement: 'This work was supported by Cancer Research UK grant number C54622/A20485'.

Competing Interest Statement: Sharon Cox has provided consultancy work for the Pacific Life insurance group on smoking and reduced risk product use and prevalence rates. Daniel Frings and Ian Albery are both investigators on a randomised controlled trial funded by Allen Carrs Easyway Ltd. This trial is comparing the Allen Carr Easway stop-smoking method to local NHS 1-1 stop smoking counselling service (ISRCTN23584477). The team are contractually free to publish the results regardless of the study outcome. They have no other conflicts of interest to declare.

Authors' contributions: Dr Frings and Prof. Albery conceptualized and designed the study and critically reviewed and revised the manuscript; Dr Cox, critically reviewed and revised the manuscript; Dr Booth drafted the initial manuscript and administered the testing, data collection and statistical analysis. All authors approved the final manuscript as submitted.

Data sharing statement: Extra data is available by emailing p.booth@uel.ac.uk or fringsd@lsbu.ac.uk

For peer review only

TREND Statement Checklist

Paper Section/ Topic	Item No	Descriptor	Reported? ✓	Pg #
**Title and Abstract**				
Title and Abstract	1	• Information on how unit were allocated to interventions		7
		• Structured abstract recommended		2
		• Information on target population or study sample		6
**Introduction**				
Background	2	• Scientific background and explanation of rationale		3-5
		• Theories used in designing behavioral interventions		7
**Methods**				
Participants	3	• Eligibility criteria for participants, including criteria at different levels in recruitment/sampling plan (e.g., cities, clinics, subjects)		6
		• Method of recruitment (e.g., referral, self-selection), including the sampling method if a systematic sampling plan was implemented		6
		• Recruitment setting		6
		• Settings and locations where the data were collected		6
Interventions	4	• Details of the interventions intended for each study condition and how and when they were actually administered, specifically including:		
		○ Content: what was given?		7
		○ Delivery method: how was the content given?		7
		○ Unit of delivery: how were the subjects grouped during delivery?		7
		○ Deliverer: who delivered the intervention?		6
		○ Setting: where was the intervention delivered?		6
		○ Exposure quantity and duration: how many sessions or episodes or events were intended to be delivered? How long were they intended to last?		6
		○ Time span: how long was it intended to take to deliver the intervention to each unit?		7
		○ Activities to increase compliance or adherence (e.g., incentives)		6
Objectives	5	• Specific objectives and hypotheses		5
Outcomes	6	• Clearly defined primary and secondary outcome measures		7
		• Methods used to collect data and any methods used to enhance the quality of measurements		6
		• Information on validated instruments such as psychometric and biometric properties		7
Sample Size	7	• How sample size was determined and, when applicable, explanation of any interim analyses and stopping rules		6
Assignment Method	8	• Unit of assignment (the unit being assigned to study condition, e.g., individual, group, community)		6
		• Method used to assign units to study conditions, including details of any restriction (e.g., blocking, stratification, minimization)		6
		• Inclusion of aspects employed to help minimize potential bias induced due to non-randomization (e.g., matching)		N/A

For peer review only

TREND Statement Checklist

Blinding (masking)	9	• Whether or not participants, those administering the interventions, and those assessing the outcomes were blinded to study condition assignment; if so, statement regarding how the blinding was accomplished and how it was assessed.		
Unit of Analysis	10	• Description of the smallest unit that is being analyzed to assess intervention effects (e.g., individual, group, or community)		
		• If the unit of analysis differs from the unit of assignment, the analytical method used to account for this (e.g., adjusting the standard error estimates by the design effect or using multilevel analysis)		
Statistical Methods	11	• Statistical methods used to compare study groups for primary methods outcome(s), including complex methods of correlated data		
		• Statistical methods used for additional analyses, such as a subgroup analyses and adjusted analysis		
		• Methods for imputing missing data, if used		
		• Statistical software or programs used		
**Results**				
Participant flow	12	• Flow of participants through each stage of the study: enrollment, assignment, allocation, and intervention exposure, follow-up, analysis (a diagram is strongly recommended)		
		○ Enrollment: the numbers of participants screened for eligibility, found to be eligible or not eligible, declined to be enrolled, and enrolled in the study		
		○ Assignment: the numbers of participants assigned to a study condition		
		○ Allocation and intervention exposure: the number of participants assigned to each study condition and the number of participants who received each intervention		
		○ Follow-up: the number of participants who completed the follow-up or did not complete the follow-up (i.e., lost to follow-up), by study condition		
		○ Analysis: the number of participants included in or excluded from the main analysis, by study condition		
		• Description of protocol deviations from study as planned, along with reasons		
Recruitment	13	• Dates defining the periods of recruitment and follow-up		
Baseline Data	14	• Baseline demographic and clinical characteristics of participants in each study condition		
		• Baseline characteristics for each study condition relevant to specific disease prevention research		
		• Baseline comparisons of those lost to follow-up and those retained, overall and by study condition		
		• Comparison between study population at baseline and target population of interest		
Baseline equivalence	15	• Data on study group equivalence at baseline and statistical methods used to control for baseline differences		

For peer review only

TREND Statement Checklist

Numbers analyzed	16	• Number of participants (denominator) included in each analysis for each study condition, particularly when the denominators change for different outcomes; statement of the results in absolute numbers when feasible		
		• Indication of whether the analysis strategy was "intention to treat" or, if not, description of how non-compliers were treated in the analyses		
Outcomes and estimation	17	• For each primary and secondary outcome, a summary of results for each estimation study condition, and the estimated effect size and a confidence interval to indicate the precision		
		• Inclusion of null and negative findings		
		• Inclusion of results from testing pre-specified causal pathways through which the intervention was intended to operate, if any		
Ancillary analyses	18	• Summary of other analyses performed, including subgroup or restricted analyses, indicating which are pre-specified or exploratory		
Adverse events	19	• Summary of all important adverse events or unintended effects in each study condition (including summary measures, effect size estimates, and confidence intervals)		
**DISCUSSION**				
Interpretation	20	• Interpretation of the results, taking into account study hypotheses, sources of potential bias, imprecision of measures, multiplicative analyses, and other limitations or weaknesses of the study		
		• Discussion of results taking into account the mechanism by which the intervention was intended to work (causal pathways) or alternative mechanisms or explanations		
		• Discussion of the success of and barriers to implementing the intervention, fidelity of implementation		
		• Discussion of research, programmatic, or policy implications		
Generalizability	21	• Generalizability (external validity) of the trial findings, taking into account the study population, the characteristics of the intervention, length of follow-up, incentives, compliance rates, specific sites/settings involved in the study, and other contextual issues		
Overall Evidence	22	• General interpretation of the results in the context of current evidence and current theory		

From: Des Jarlais, D. C., Lyles, C., Crepaz, N., & the Trend Group (2004). Improving the reporting quality of nonrandomized evaluations of behavioral and public health interventions: The TREND statement. *American Journal of Public Health*, 94, 361-366. For more information, visit: http://www.cdc.gov/trendstatement/

For peer review only

BMJ Open

The effect of viewing an on-line e-cigarette advertisement on attitudes towards cigarette and e-cigarette use: a cross-sectional study

Journal:	*BMJ Open*
Manuscript ID	bmjopen-2018-027525.R1
Article Type:	Research
Date Submitted by the Author:	25-Mar-2019

Complete List of Authors:	Booth, Paula; University of East London, Psychology<br>Albery, Ian; London South Bank University Department of Applied Sciences, Division of Psychology (School of Applied Sciences)<br>Cox, Sharon; London South Bank University Department of Applied Sciences, Division of Psychology (School of Applied Sciences)<br>Frings, Daniel; London South Bank University Department of Applied Sciences, Division of Psychology (School of Applied Sciences)

**Primary Subject Heading**:	Public health
Secondary Subject Heading:	Smoking and tobacco, Public health
Keywords:	attitude, advertisement, e-cigarettes, tobacco, dual-users, smokers

SCHOLARONE™
Manuscripts

The effect of viewing an on-line e-cigarette advertisement on attitudes towards cigarette and e-cigarette use: a cross-sectional study

Paula Booth[1], Ian. P. Albery[2], Sharon Cox[2] & Daniel Frings[2]

[1](Paula Booth – corresponding author) Applied Health and Communities College, School of Psychology, Psychological Sciences Department, The University of East London, Stratford Campus, Water Lane

London, E15 4LZ. p.booth@uel.ac.uk

Email: p.booth@uel.ac.uk

Phone: +44 (0)20 8223 4719

[2]Division of Psychology, School of Applied Sciences, London South Bank University, 103 Borough Road, London, SE1 0AA

Key words: E-Cigarette; Tobacco; Attitudes; Advertisement; Dual-Users

Word Count : 3822

For peer review only

Objectives: This study explored the potential for e-cigarette advertisements to (i) enhance attitudes towards cigarettes and/or (ii) reduce barriers to e-cigarettes uptake. The study tested whether exposure to an on-line electronic cigarette advertisement changed attitudes towards cigarettes and e-cigarettes in smokers, non-smokers, e-cigarette users and dual-users (smokers who also use e-cigarettes).

Design: Cross-sectional study

Setting: Online survey

Participants: Adults (n=964) aged 18 to 65 years old (*M* = 36 years, *SD* = 11.6) from the UK and USA. Participants were grouped into current non-smokers, e-cigarette users, dual-users and smokers.

Interventions: Participants viewed one of 15 randomly assigned online e-cigarette advertisements.

Primary measures: Three single 7 point Likert scales measuring health, desirability, social acceptability were completed pre and post advertisement exposure.

Results: Post exposure all smoking groups showed a decrease or no change in how socially acceptable or desirable they rated cigarettes. Paradoxically, dual-users rated cigarettes as being significantly healthier after viewing the advertisement (p =.01) whilst all other smoking group ratings remained the same. There was an increase or no change in how all smoking groups perceived the healthiness and desirability of e-cigarettes

Conclusions: We observed no evidence that exposure to an e-cigarette advertisement renormalizes or encourages smoking in smokers, non-smokers or e-cigarette users. However, there is some indication that viewing an e-cigarette advertisement may increase duals-users' perceptions of the health of smoking.

For peer review only

Strengths and limitations of this study

- This study is among the first to examine the attitudinal processes underlying the effect of viewing an advertisement on interest in both smoking and vaping.
- The sample were derived of smokers, non-smokers, vapers and dual users rather than focusing on only one smoking type which is often a limitation of the extant literature in this area of research.
- The effects of viewing an advertisement on actual smoking behaviour was not assessed and should be considered in future work.
- Multiple analyses increased the risk of Type 1 errors.
- The effects of different modalities of advertising were not assessed.

For peer review only

INTRODUCTION

There is a large body of evidence to support the notion that advertising cigarettes encourages people to start or to continue to smoke.[1,2] With the intent of reducing tobacco use globally, Article 13 of the WHO Framework Convention for Tobacco Control calls for "a comprehensive ban on advertising, promotion and sponsorship of cigarettes"[3] (pg. 11). However, this treaty does not cover e-cigarettes. While there are restrictions on advertising e-cigarettes in the UK (Committee on Advertising Practice)[4] and in the USA (U.S. Food and Drug Regulations)[5] e-cigarettes are still being marketed elsewhere and there is concern that the advertisements portray e-cigarettes as being glamourous.[6] In these territories the public now experiences increased exposure to e-cigarette advertisements[4,7,8] in the form of television, magazines, newspapers, billboards and internet advertisements.[9] The current paper explores the potential of such advertisements to (i) enhance attitudes towards cigarettes and (ii) reduce barriers to e-cigarette uptake amongst current smokers.

One concern related to e-cigarette advertisements is that they may renormalize smoking. The de-normalisation of smoking has been a cornerstone of tobacco control policies in encouraging existing smokers to quit and also to discourage initial uptake[10–12] and social acceptance of smoking has been shown to be a barrier to reduction in smoking prevalence in a variety of communities.[13,14] It is therefore important to determine whether e-cigarette advertising may also affect attitudes towards cigarettes by making them more positive. A number of researchers have suggested that e-cigarette advertising could lead to increases in how socially acceptable and desirable cigarettes are perceived to be, and subsequently influence their continued use or possible (re)uptake in smokers, e-cigarette users and dual-users (those who use both cigarettes and e-cigarettes).[6,15–17]. Alongside observed effects on these groups, concerns that e-cigarette use may be a gateway to smoking suggests it is also

For peer review only

important to determine whether advertising may encourage non-smokers to use e-cigarettes.[16,17] In this context, it is important to note that the evidence base around actual usage statistics currently suggests that e-cigarette availability does not appear to have led to regular use of either e-cigarettes or smoking amongst British non-smokers: the current number of UK non-smokers using an e-cigarette daily is less than 1%, and smoking prevalence rates continue to decline.[18] A similar pattern is observed in the US.[19] However, results from laboratory studies exploring the effects of advertisements on urges to smoke and interest in smoking amongst other groups are inconsistent, and none have examined attitudinal processes which may underlie those effects which have been observed.[20–22] The current study aims to examine the issue of renormalisation by measuring attitude change towards cigarettes in adult non-smokers, smokers, e-cigarette users and dual-users before and after viewing an advertisement.

In contrast to concerns about renormalisation, e-cigarette advertisements may represent an opportunity to promote reduced risk products as an alternative to smoking. Although there are currently only relatively few studies comparing the health effects of e-cigarettes use to smoking or non-smoking over time,[23,24] in the UK e-cigarettes are widely supported as a reduced risk nicotine alternative for smokers, and has received support from a number of agencies. [18,25–27] However in the UK, despite increasing public agency support, one barrier to the uptake of e-cigarettes amongst smokers has been an increasingly negative attitudes towards the products. Between 2015 and 2017 the proportion of UK smokers who believed that e-cigarettes are less harmful than cigarettes reduced from 31% to 20% (Action on Smoking and Health)[27]. Similarly, in the USA the percentage of smokers who perceived e-cigarettes to be equally harmful than combustible cigarettes increased from 23% in 2012 to 35% in 2015.[28] To the extent attitudes predict behaviour, such negative perceptions of the health of e-cigarettes are likely to reduce the potential for e-cigarettes to be used as a smoking

For peer review only

cessation aid. E-cigarette advertising may serve to halt or reverse this decline. Research examining the effects of advertising on perception of e-cigarettes (carried out predominantly on younger populations) shows that viewing an advertisement may increase the perception of social acceptability and the intention to try an e-cigarette in the future.[7,29] For instance, 13-17 year old smokers showed a more positive attitude towards e-cigarettes after viewing an e-cigarette advertisement,[29] and adult smokers reported a decrease in the acute urge to smoke[30] and an interest in trying e-cigarettes.[31–33] Thus, in the current study we also explore the potential for e-cigarette advertisements to change the way smokers (and other groups) perceive the healthiness, socially acceptability and general desirability of e-cigarettes.[13,34]

One limitation of the extant literature in these areas is that it focuses on either smokers, non-smokers and/or e-cigarette users. This leaves one important population, dual-users, understudied. In a survey of e-cigarette use in Europe, of an estimated 37 million e-cigarette users, 73% were currently still smoking.[35] Many dual-users use e-cigarettes in areas in which they are unable to smoke cigarettes.[36] However, they still have a preference for smoking cigarettes in stressful situations or for pleasure.[37] Thus, it is important to establish whether viewing an e-cigarette advertisement can change attitudes towards smoking in dual-users.

To explore the potential for-cigarette advertisements to (i) enhance attitudes towards cigarettes and/or (ii) reduce barriers to e-cigarettes uptake, the current study investigated whether viewing an e-cigarette advertisement influenced the extent to which smoking and e-cigarettes were perceived to be more or less healthy, desirable and socially acceptable in dual-users, smokers, non-smokers and e-cigarette users.

METHODS

For peer review only

Design

A cross sectional design in which attitude measures were taken pre and post advertisement viewing. The between-subjects factor was smoking group (non-smokers, e-cigarette users, dual-users and smokers). The dependent variables were the scores on a series of questions rating attitudes around the healthiness, desirability and social acceptability of both e-cigarettes and cigarettes.

Participants, Recruitment and Procedure

Originally, 964 participants completed the survey, between December 2015 and February 2016, but data from 199 were removed for being under the age of 18 or not entering their age, failing controls (see below) and having missing data. Data were included for 765 participants, 361 male, 400 female and 4 others from an age range of 18-65 years old (M = 36 years, SD = 11.6). Participants were defined as smokers (n=115), non-smokers (410), e-cigarette users (100) or dual-users (145). They were located in the USA (n=543) or the UK (n=222).

Participants were recruited through an online crowd-sourcing tool (Crowdflower, similar to MTurk). Crowdflower users were given information about the survey on the Crowdflower site before being given the opportunity to proceed by giving online consent to participate in the study and then clicking on the link to be transferred to a survey (delivered using Qualtrics). Participants were paid a fee of $1.00 to complete the survey. Control questions were built into the Qualtrics survey to exclude automated responses and participants who were not concentrating appropriately on the questions (for example, “For this item please indicate strongly agree”). Additional controls were set up on the survey so that participants were unable to enter the site from the same IP address more than once and that they had to take a minimum time of 90 seconds to complete the survey. Post-hoc analysis indicated that a

For peer review only

minimum of 100 participants (the smallest smoking group) was sufficient to detect an effect size r =.15 or greater, (with Z = 2.2 or above).

Patient and public involvement.

Patients were not involved in the development or conduct of this study. There are plans to disseminate the results to practitioners by including the results as an aspect in London South Bank courses provision aimed at service delivery managers and counsellors. A summary of the findings will be offered to Cancer Research UK and other policy organisations and promoted on relevant staff social media sites.

Measures

Attitudes towards Cigarettes

Participants rated on three single measures how *healthy*, how *desirable* and how *socially acceptable* they found cigarettes and e- cigarettes on a 7-point Likert type scale ranging from 1 '*strongly disagree'* to 7 '*strongly agree*.' They were given two separate statements: "When thinking about tobacco/e- cigarettes please indicate whether you think they are……." The order in which e-cigarettes and tobacco cigarette statements were presented were counter-balanced to reduce question order bias.

Smoking Status

Participants were asked to indicate how often (never, occasionally, very often, always) they smoked, vaped or used nicotine products both in the present and the past. Participants were placed in smoking groups based on their 'present' smoking status. Those that answered 'never' to using tobacco or e-cigarettes were defined as non-smokers, those that answered 'occasionally', 'very often' or 'always' were defined as smokers, e-cigarette users or dual-users as appropriate.

Procedure

For peer review only

The study was given ethical approval by the London South Bank University Research Ethics Committee (UREC1564). The data reported here were used to identify which advertisement would be used in a larger study. The current study and the larger study were funded by Cancer Research UK (CRUK grant number C54622/A20485).

Participants were asked to rate how healthy, desirable and socially acceptable they found tobacco and e-cigarettes before and after viewing one of fifteen e-cigarette advertisements (see below). In addition, after completing the post-test attitude questions they were asked to rate the emotional attributes and the perceived effectiveness of the advertisement and to give demographic characteristics. Finally, they were given a debrief about the study, a warning about the addictive nature of nicotine and given links to government quit smoking websites.

Advertisement Selection and Preparation

Each participant viewed one of fifteen advertisements which were allocated randomly in Qualtrics. The fifteen advertisements were chosen from a pool of 200 different advertisements displayed online between 2013 and 2016. Ten different themes of advertising were identified which depicted e-cigarettes as being; a smoking cessation tool, healthier than (tobacco) cigarettes, aesthetically pleasing, celebrity endorsed, sporty, an alternative to cigarettes in places where cigarettes were restricted, as satisfying, cheaper, more fragrant and as cool as cigarettes.[6] Five researchers coded each advertisement as one of the ten themes and advertisements were chosen that were consistently coded as the same theme by three of the five coders and which the research team found to be the most engaging. The final 15 advertisements included all ten themes, a variety of brands and images[31] and eight included a smoking or vaping cue and seven did not.[20]

RESULTS

For peer review only

Observation of Table 1 show that only 2.9% of non-smokers had been full-time smokers in the past and none had been full-time vapers. Only 10% of current e-cigarette users had not smoked in the past; most were either full-time or intermittent smokers previously. There were no smokers who had transferred from daily e-cigarette use to smoking, although 27.8% had used e-cigarettes intermittently in the past. Most dual-users had smoked or used e-cigarettes intermittently in the past.

Table 1. Percentage of previous tobacco and e-cigarette use of current non-smokers, e-cigarette users, smokers and dual-users.

Previous Use	*Status*	Current Non-smokers	Current E-cigarette User	Current Smoker	Current Dual-User
Tobacco Use	Never	68.5%	10.0%	0.9%	0.7%
	Intermittent	28.5%	49%	67.8%	72.9%
	Always	2.9%	41%	31.3%	26.4%
E-cigarette Use	Never	93.9%	21%	72.2%	5.7%
	Intermittent	6.1%	50%	27.8%	88.7%
	Always	0.0%	29%	0.0%	3.6%

Note: The categories 'occasionally' and 'very often' were collapsed and named intermittent

Health scores in the smokers group were not normally distributed due to the positive skew (baseline skewness = 2.63, test skewness = 2.40) thus Wilcoxon Signed Rank Tests were used to compare pre and post intervention scores in each smoking group (smokers, non-smokers, dual-users and e-cigarette users) (see Table 2).

Table 2. Mean healthy, desirability and social acceptability ratings (standard deviation in parentheses) towards tobacco and e-cigarettes by smoking group pre and post advertisement presentation.

		Sample							
Attitude target	*Attitude dimension*	Non-smoker (n=410)		E-cigarette user (n=100)		Smoker (n=115)		Dual-User (n= 145)	
		Pre	Post	Pre	Post	Pre	Post	Pre	Post
CigaretteCigarette	Healthy	1.27 (0.83)	1.28 (0.76)	1.47 (0.90)	1.47 (0.81)	1.59 (0.90)	1.73 (1.03)	2.08 (1.35)	**2.26**** **(1.50)**
	Desirability	1.86 (1.45)	1.77 (1.35)	2.84 (1.79)	**2.58*** **(1.67)**	3.52 (1.78)	**3.21**** **(1.67)**	3.85 (1.87)	3.71 (1.82)
	Social Acceptability	2.63 (1.57)	**2.39***** **(1.54)**	3.04 (1.76)	**2.86*** **(1.78)**	3.43 (1.55)	3.30 (1.64)	3.36 (1.67)	3.39 (1.77)
E-Cigarette	Healthy	2.53 (1.38)	**2.68**** **(1.51)**	4.79 (1.60)	4.82 (1.65)	3.03 (1.48)	**3.21***** **(1.54)**	3.80 (1.33)	**4.01**** **(1.42)**
	Desirability	2.76 (1.57)	**2.91*** **(1.70)**	5.36 (1.44)	5.21 (1.37)	3.91 (1.45)	3.91 (1.54)	4.82 (1.21)	**5.03**** **(1.24)**
	Social Acceptability	3.93 (1.58)	**3.81*** **(1.07)**	5.08 (1.45)	5.07 (1.46)	4.59 (1.39)	4.50 (1.40)	5.28 (1.05)	5.14 (1.18)

Note: Comparisons are made pre-post for each dimension for each group, with significant differences marked with asterisk(es): * = p<0.05 **= p<0.005 ***= p<0.001

For peer review only

Attitudes Towards Cigarettes

All smoking groups gave low scores for health of cigarettes at baseline. Showing an e-cigarette advertisement made no significant difference to the score in the smoking, non-smoking and e-cigarette user groups but dual-users scored cigarettes as healthier after viewing the advertisement (Z = 2.57 p =.01). There was no change in dual-users rating of desirability or social acceptability after viewing an advertisement. E-cigarette users scored cigarettes as significantly less desirable (Z = -2.50, p =.013) and less socially acceptable (Z = -2.501 p =.012) after viewing an advertisement. Smokers scored cigarettes as less desirable (Z = -2.81, p =.005) after viewing an advertisement but there was no difference in pre and post scores of social acceptability. Non-smokers showed no change in desirability scores of

cigarettes but scored them as less socially acceptable (Z = -4.67 p >.001) after viewing an advertisement.

Non-smokers scored e-cigarettes as being healthier after viewing an e-cigarette advertisement (Z = 2.97, p =.003), more desirable (Z = 2.60, p =.009) but less socially acceptable (Z = -2.12 p =.034). E-cigarette users showed no change in attitudes. Smokers scored e-cigarettes as healthier after viewing the advertisements (Z = 2.21, p =.027) but there was no change in desirability or social acceptability. Dual-users scored e-cigarettes as being healthier after viewing advertisement (Z = 2.53, p =.011) and more desirable (Z = 2.04, p =.042) but there was no change in social acceptability.

DISCUSSION

The current study aimed to explore the potential of e-cigarette advertising to (i) enhance attitudes towards cigarettes by reducing negative evaluations towards them and (ii) reduce barriers to the uptake of e-cigarettes by smokers. In terms of renormalisation by changes in attitude, little evidence in the current study supports the notion that viewing e-cigarette advertisements increased positive evaluations of cigarettes. Rather, perceptions of cigarettes being socially acceptable and desirable generally decreased. All smoking groups, other than dual-users, showed no change in health scores for cigarettes. However, these groups gave scores close to the scale's floor for health at baseline and there may have been no possibility to reduce scores further after viewing the advertisement. Thus, there was no indication that viewing an e-cigarette advertisement renormalized or encouraged smoking amongst these smokers, non-smokers and e-cigarette users.

After viewing an advertisement smokers scored cigarettes as less desirable and e-cigarettes as healthier supporting the notion that advertisements may reduce barriers to the uptake of e-cigarettes by smokers. Indeed, all groups scored e-cigarettes as healthier after viewing the

For peer review only

advertisement, other than e-cigarette users, whose baseline scores were almost at ceiling already. These results show that e-cigarette advertising successfully increases positive attitudes towards e-cigarettes. As perceptions of health are a key determinant in behaviour change these results encourage the view that smokers may potentially use e-cigarettes as a tool for smoking cessation.[38]

In non-smokers scores for social acceptability of both cigarettes and e-cigarettes decreased. This result is inconsistent with previous findings which found that when young people viewed e-cigarette advertisements the ratings for social acceptability increased.[7,29] This discrepancy in results may be due to the different age groups being tested and the suggestion that e-cigarette adverts (and products) are often designed with a younger target audience in mind.[39] It also mirrors a more general negative attitude towards e-cigarettes observed in the UK population in recent years.[27] However, in the current study, after viewing the advertisement the perception that e-cigarettes are healthy and desirable increased amongst non-smokers. Although widespread use of e-cigarettes by non-smokers has not been observed to date (in populations where data is available), this result suggests the effects of e-cigarettes advertising on actual e-cigarettes uptake amongst nicotine naïve users should continue to be monitored, especially in territories where advertising is prevalent.

For peer review only

E-cigarette users showed no change in attitudes towards e-cigarettes after viewing an advertisement. Perceptions of desirability and social acceptability of cigarettes decreased after watching the advertisement. A large proportion of e-cigarette users had previously been smokers so these findings support the view that viewing an e-cigarette advertisement may help deter ex-smokers, who are vaping as a smoking cessation tool, from relapsing.

One group who are relatively under-researched (dual-users) displayed a different pattern of responses to cigarettes to the other smoking categories. They rated cigarettes and e-cigarettes

as healthier after viewing the advertisement. The contradictory effect of the advertisements on the attitudes of this particular group suggests that the drive to use both tobacco and e-cigarettes is complex. Indeed, some research suggests that many dual-users may still use cigarettes because they find it to be a pleasurable experience and use e-cigarettes as a practical solution when smoking cigarettes is banned.[37] Although our initial explorations of this group warrant caution in their interpretation, they do flag the need for further research exploring how dual-users perceive and value the differences between e-cigarettes and smoking, and to identify the best strategy to help motivate this group to achieve cessation from combustible products.

Study Limitations

This was an exploratory study and we acknowledge that there were some limitations but that the current work can still contribute to advances in knowledge in the field. As the general public are likely to view a wide range of e-cigarette advertisement types, themes and categories online we purposely chose to measure changes in attitudes to a variety of advertisements across broad smoking categories rather than assessing the effects of different types of e-cigarette advertisement, for example cue versus no cue, on specific smoking status. Future research could assess the effects of different types of e-cigarette advertisement on specific smoking status, for example daily versus intermittent smokers and vapers.

Due to the non-parametric nature of the data multiple analyses were carried out and thus there was an increased risk of Type 1 errors. After deliberation we chose not to use a statistical method to correct the experiment-wise error rate as this correction may then have increased the risk for Type 2 errors. As the purpose of this study was to explore the data with a view to providing a baseline from which to replicate the findings and inform further studies we

For peer review only

considered that making a correction may hinder the accumulation of knowledge in this area and that increased Type 1 errors were preferential to increased Type 2 errors.[40,41]

In this study we measured attitudes towards cigarettes and e-cigarettes but did not assess any effects on intention to smoke or actual smoking behaviour. Recent evidence shows that viewing an e-cigarette product[42] or advertisement[43] may act as a cue that leads to an increased urge and desire to smoke or vape. Therefore, future research needs to investigate the relationship between attitude and cue reactivity. The current study had a cross-sectional design and the change in attitude was measured immediately after one exposure to the advertisement so it cannot be determined whether effects of viewing an advertisement would persist over a longer time period. Future research would benefit from assessing changes in attitude longitudinally as well as measuring intentions to smoke or use e-cigarettes and actual smoking behaviour for a fixed period before and after viewing the advertisement.

Our outcomes used single measure attitude scales and although these had high external validity (and single item scales can be regarded as appropriate when constructs are well defined),[44] multiple items scales would have allowed empirical assessment of validity. Furthermore, measuring attitudes towards health using a seven-point Likert type scale resulted in possible floor effects. A more sensitive measure such as a Visual Analogue Scale may have given a broader range of data ensuring that the data was more normally distributed and giving a better indication of changes that may have occurred at the lower end of the scale.

There is a social stigma associated with smoking and so there may have been an effect of social desirability bias. Attitudes in the current study were assessed using explicit, self-report measures so an implicit measure of attitude could be measured in future research as previous research shows that these measures of attitude interact to predict actual smoking behaviour.[45] Additionally, it was considered whether results may be a consequence of the

For peer review only

boomerang effect. The advertisements trying to persuade e-cigarettes to be viewed more positively may have inadvertently caused the consumer to resist the persuasion attempt and instead view e-cigarettes more negatively or cigarettes more positively, as a form of non-compliance. Results were not unexpected, other than for dual-users, and so the use of a control group in future research may help to elucidate more of an understanding of attitude changes in response to advertisements in this particular group.

Conclusion

The results from this study suggest that viewing an e-cigarette advertisement is unlikely to renormalize smoking amongst most groups. However, we observed some evidence that e-cigarette advertisements may increase how healthy cigarettes are perceived amongst dual-users. This highlights the importance of more research with this relatively understudied group, in particular around factors which promote the decision to transition from dual-use to e-cigarette use or nicotine abstinence. In terms of reducing barriers to uptake, e-cigarette advertisement may encourage smokers to quit using e-cigarettes as a smoking cessation tool. Thus, e-cigarette advertisements could be considered a viable tool to stimulate or support smoking cessation. However, where e-cigarette advertising is prevalent, careful monitoring of uptake of e-cigarettes amongst nicotine naïve populations is important.

For peer review only

References

1 Evans N, Farkas A, Gilpin E, *et al.* Influence of Tobacco Marketing and Exposure to Smokers on Adolescent Susceptibility to Smoking. *JNCI J Natl Cancer Inst* 1995;**87**:1538–45. doi:10.1093/jnci/87.20.1538

2 Tye JB, Warner KE, Glantz SA. Tobacco Advertising and Consumption: Evidence of a Causal Relationship. *J Public Health Policy* 1987;**8**:492. doi:10.2307/3342275

3 WHO FRAMEWORK CONVENTION ON TOBACCO CONTROL WHO Library Cataloguing-in-Publication Data. Published Online First: 2010.http://apps.who.int/iris/bitstream/handle/10665/42811/9241591013.pdf;jsessionid=62CFB43C3F37022BD16CEA88E7D3DF93?sequence=1 (accessed 16 Apr2018).

4 New rules for the marketing of e-cigarettes CAP and BCAP's Joint Regulatory Statement. https://www.asa.org.uk/asset/851604F6-C081-420C-9DDDA65124B775A4/ (accessed 21 Aug2017).

5 Commissioner O of the. Economic Impact Analyses of FDA Regulations - Summary: Deeming Tobacco Products to be Subject to the Food, Drug, and Cosmetic Act, as Amended by the Family Smoking Prevention and Tobacco Control Act; Regulations Restricting the Sale and Distribution of Tobacco Products and Required Warning Statements for Tobacco Product Packages and Advertisements (Final Rule). https://www.fda.gov/AboutFDA/ReportsManualsForms/Reports/EconomicAnalyses/ucm500249.htm (accessed 16 Apr2018).

6 De Andrade M, Hastings G, Angus K, *et al.* the marketing of electronic cigarettes in the uk. Published Online First: 2013.https://dspace.stir.ac.uk/bitstream/1893/17889/1/deAndradeetale-cigsreport.pdf (accessed 26 Mar2017).

7 Duke JC, Lee YO, Kim AE, *et al.* Exposure to Electronic Cigarette Television Advertisements Among Youth and Young Adults. *Pediatrics* 2014;**134**.http://pediatrics.aappublications.org/content/134/1/e29.full (accessed 21 Aug2017).

8 Basch CH, Mongiovi J, Hillyer GC, *et al.* An Analysis of Electronic Cigarette and Cigarette Advertising in US Women's Magazines. *Int J Prev Med* 2016;**7**:103. doi:10.4103/2008-7802.190089

9 Bauld L, Angus K, De Andrade M, *et al.* Electronic Cigarette Marketing: Current Research and Policy. 2016. http://www.cancerresearchuk.org/sites/default/files/electronic_cigarette_marketing_report_final.pdf (accessed 21 Aug2017).

10 Linkenbach, J. W., & Perkins HW. MOST of us are tobacco free: An eight-month social norms campaign reducing youth initiation of smoking in Montana. In: H. W. Perkins, ed. *The social norms approach to preventing school and college age substance abuse: A handbook for educators, counselors, and clinicians*. San Francisco, CA, US: : Jossey-Bass 2003. 224–34.http://psycnet.apa.org/record/2003-02237-013 (accessed 16 Apr2018).

11 Reid JL, Manske SR, Leatherdale ST. Factors related to adolescents' estimation of peer smoking prevalence. *Health Educ Res* 2007;**23**:81–93. doi:10.1093/her/cym006

12 Bayer R, Bachynski KE. Banning Smoking In Parks And On Beaches: Science, Policy, And The Politics Of Denormalization. *Health Aff* 2013;**32**:1291–8. doi:10.1377/hlthaff.2012.1022

13 Nicholson AK, Borland R, van der Sterren AE, *et al.* Social acceptability and desirability of smoking in a national sample of Aboriginal and Torres Strait Islander people. *Med J Aust* 2015;**202**:57–62. doi:10.5694/mja14.01534

14 Twyman L, Bonevski B, Paul C, *et al.* Perceived barriers to smoking cessation in selected vulnerable groups: a systematic review of the qualitative and quantitative literature. *BMJ Open* 2014;**4**:e006414. doi:10.1136/bmjopen-2014-006414

15 Fairchild AL, Bayer R, Colgrove J. The Renormalization of Smoking? E-Cigarettes and the Tobacco "Endgame". *N Engl J Med* 2014;**370**:293–5. doi:10.1056/NEJMp1313940

16 Schneider S, Diehl K. Vaping as a Catalyst for Smoking? An Initial Model on the Initiation of Electronic Cigarette Use and the Transition to Tobacco Smoking Among Adolescents. *Nicotine Tob Res* 2016;**18**:647–53. doi:10.1093/ntr/ntv193

17 Etter J-F. Gateway effects and electronic cigarettes. *Addiction* Published Online First: 7 August 2017. doi:10.1111/add.13924

For peer review only

18 Mcneill A, Brose LS, Calder R, *et al.* Evidence review of e-cigarettes and heated tobacco products 2018 A report commissioned by Public Health England. Published Online First: 2018.https://assets.publishing.service.gov.uk/government/uploads/system/uploads/attachment_data/file/684963/Evidence_review_of_e-cigarettes_and_heated_tobacco_products_2018.pdf (accessed 16 Apr2018).
19 *QuickStats:* Cigarette Smoking Status* Among Current Adult E-cigarette Users,† by Age Group — National Health Interview Survey,§ United States, 2015. *MMWR Morb Mortal Wkly Rep* 2016;**65**:1177. doi:10.15585/mmwr.mm6542a7
20 Maloney EK, Cappella JN. Does Vaping in E-Cigarette Advertisements Affect Tobacco Smoking Urge, Intentions, and Perceptions in Daily, Intermittent, and Former Smokers? *Health Commun* 2015;:1–10.
21 Durkin SJ, Bayly M, Wakefield MA. Can E-cigarette Ads Undermine Former Smokers? An Experimental Study. *Tob Regul Sci* 2016;**2**:263–77. doi:10.18001/TRS.2.3.6
22 Smith DM, Bansal-Travers M, O'Connor RJ, *et al.* Associations between perceptions of e-cigarette advertising and interest in product trial amongst US adult smokers and non-smokers: results from an internet-based pilot survey. *Tob Induc Dis* 2015;**13**:14. doi:10.1186/s12971-015-0039-6
23 Shahab L, Goniewicz ML, Blount BC, *et al.* Nicotine, Carcinogen, and Toxin Exposure in Long-Term E-Cigarette and Nicotine Replacement Therapy Users. *Ann Intern Med* 2017;**166**:390. doi:10.7326/M16-1107
24 Polosa R, Cibella F, Caponnetto P, *et al.* Health impact of E-cigarettes: a prospective 3.5-year study of regular daily users who have never smoked. *Sci Rep* 2017;**7**:13825. doi:10.1038/s41598-017-14043-2
25 E-cigarettes | Cancer Research UK. http://www.cancerresearchuk.org/about-cancer/causes-of-cancer/smoking-and-cancer/e-cigarettes (accessed 16 Apr2018).
26 Royal College of Physicians. Nicotine without smoke: Tobacco harm reduction | RCP London. https://www.rcplondon.ac.uk/projects/outputs/nicotine-without-smoke-tobacco-harm-reduction-0/ (accessed 18 Dec2017).
27 ASH Action on Smoking and Health. Use of electronic cigarettes (vapourisers) among adults in Great Britain. ASH Fact Sheet 2017. 2017.
28 Majeed B, Weaver S, … KG-A journal of, *et al.* Changing perceptions of harm of e-cigarettes among US adults, 2012–2015. *Elsevier*https://scholar.google.co.uk/scholar?hl=en&as_sdt=0%2C5&q=Changing+perceptions+of+harm+of+e-cigarettes+among+US+adults%2C+2012–2015&btnG= (accessed 16 Apr2018).
29 Farrelly MC, Duke JC, Crankshaw EC, *et al.* A Randomized Trial of the Effect of E-cigarette TV Advertisements on Intentions to Use E-cigarettes. *Am J Prev Med* 2015;**49**:686–93. doi:10.1016/j.amepre.2015.05.010
30 Jo CL, Golden SD, Noar SM, *et al.* Effects of E-cigarette Advertising Messages and Cues on Cessation Outcomes. *Tob Regul Sci* 2018;**4**:562–72. doi:10.18001/TRS.4.1.3
31 Pepper JK, Brewer NT. Electronic nicotine delivery system (electronic cigarette) awareness, use, reactions and beliefs: a systematic review. *Tob Control* 2014;**23**:375–84. doi:10.1136/tobaccocontrol-2013-051122
32 Kim AE, Lee YO, Shafer P, *et al.* Adult smokers' receptivity to a television advert for electronic nicotine delivery systems. *Tob Control* 2015;**24**:132–5. doi:10.1136/tobaccocontrol-2013-051130
33 Smith DM, Bansal-Travers M, O'Connor RJ, *et al.* Associations between perceptions of e-cigarette advertising and interest in product trial amongst US adult smokers and

For peer review only

non-smokers: results from an internet-based pilot survey. *Tob Induc Dis* 2015;**13**:14. doi:10.1186/s12971-015-0039-6
34 Chapman S, Freeman B. Markers of the denormalisation of smoking and the tobacco industry. *Tob Control* 2008;**17**:25–31. doi:10.1136/tc.2007.021386
35 Farsalinos KE, Poulas K, Voudris V, *et al.* Electronic cigarette use in the European Union: analysis of a representative sample of 27 460 Europeans from 28 countries. *Addiction* 2016;**111**:2032–40. doi:10.1111/add.13506
36 Adriaens K, Van Gucht D, Baeyens F. Differences between Dual Users and Switchers Center around Vaping Behavior and Its Experiences Rather than Beliefs and Attitudes. *Int J Environ Res Public Health* 2017;**15**:12. doi:10.3390/ijerph15010012
37 Maglia M, Caponnetto P, Di Piazza J, *et al.* Dual use of electronic cigarettes and classic cigarettes: a systematic review. *Addict Res Theory* 2017;:1–9. doi:10.1080/16066359.2017.1388372
38 Webb TL, Joseph J, Yardley L, *et al.* Using the internet to promote health behavior change: a systematic review and meta-analysis of the impact of theoretical basis, use of behavior change techniques, and mode of delivery on efficacy. *J Med Internet Res* 2010;**12**:e4. doi:10.2196/jmir.1376
39 Richardson A, Ganz O, Vallone D. Tobacco on the web: surveillance and characterisation of online tobacco and e-cigarette advertising. *Tob Control* 2015;**24**:341–7. doi:10.1136/tobaccocontrol-2013-051246
40 Armstrong RA. When to use the Bonferroni correction. *Ophthalmic Physiol Opt* 2014;**34**:502–8. doi:10.1111/opo.12131
41 Nakagawa S. A farewell to Bonferroni: the problems of low statistical power and publication bias. *Behav Ecol* 2004;**15**:1044–5. doi:10.1093/beheco/arh107
42 King AC, Smith LJ, McNamara PJ, *et al.* Second Generation Electronic Nicotine Delivery System Vape Pen Exposure Generalizes as a Smoking Cue. *Nicotine Tob Res* 2017;**20**:ntw327. doi:10.1093/ntr/ntw327
43 Chen Y, Fowler CH, Papa VB, *et al.* Adolescents' behavioral and neural responses to e-cigarette advertising. *Addict Biol* 2018;**23**:761–71. doi:10.1111/adb.12510
44 Postmes T, Haslam SA, Jans L. A single-item measure of social identification: Reliability, validity, and utility. *Br J Soc Psychol* 2013;**52**:597–617. doi:10.1111/bjso.12006
45 Perugini M. Predictive models of implicit and explicit attitudes. *Br J Soc Psychol* 2005;**44**:29–45. doi:10.1348/014466604X23491

Funding statement: 'This work was supported by Cancer Research UK grant number C54622/A20485'.

Competing Interest Statement: Sharon Cox has provided consultancy work for the Pacific Life insurance group on smoking and reduced risk product use and prevalence rates. Daniel Frings and Ian Albery are both investigators on a randomised controlled trial funded by Allen Carrs Easyway Ltd. This trial is comparing the Allen Carr Easway stop-smoking method to local NHS 1-1 stop smoking counselling service (ISRCTN23584477). The team are contractually

For peer review only

free to publish the results regardless of the study outcome. They have no other conflicts of interest to declare.

Authors' contributions: Dr Frings and Prof. Albery conceptualized and designed the study and critically reviewed and revised the manuscript; Dr Cox, critically reviewed and revised the manuscript; Dr Booth drafted the initial manuscript and administered the testing, data collection and statistical analysis. All authors approved the final manuscript as submitted.

Data sharing statement: Extra data is available by emailing p.booth@uel.ac.uk or fringsd@lsbu.ac.uk

For peer review only

Survey Questions

Please indicate your gender: Male/Female/Other

Where are you based? UK/US

Please indicate your age ____

(Likert scales anchored at 1= Strongly Disagree, 7 = Strongly Agree)

Thinking about e-cigarettes, please indicate if you think they are:

Healthy

Desirable

Socially acceptable

For this item, please indicate 'Strongly Agree'*

Thinking about tobacco cigarettes

Healthy

Desirable

Socially acceptable

For this item, please indicate 'Strongly Disagree'*

Participants are then shown one of fifteen images, selected from publically available e-cigarette adverts / stop smoking campaigns).

Please indicate the extent you this image is;

(Visual Analogue Scale 0 =Not At All, 100=Very Much)

Persuasive (would it persuade you to (buy the product)

Effective

Happy

Sad

Scary

Exciting

Engaging

Are you still there? For this item, please indicate 'Very Much'*

For peer review only

How visually complex is the image?

(1 = Very simple, 10 = Very complex).

(Likert scales anchored at 1= Strongly Disagree, 7 = Strongly Agree)

Now you have seen this image and thinking about e-cigarettes, please indicate if you think they are:

Healthy

Desirable

Socially acceptable

For this item, please indicate 'Disagree'*

Now you have seen this image and thinking about tobacco cigarettes, please indicate if you think they are:

Healthy

Desirable

Socially acceptable

For this item, please indicate 'Somewhat Agree'*

** These items are 'zombie' checks which reduce the amount of automated ratings being used*

(Likert scales anchored at 1= Never, 2=Occasionally, 3=Very Often, 4=Always)

AT PRESENT, do you vape, smoke or use nicotine products?

E-Cigarettes

Tobacco Cigarettes

Nicotine Inhalers, nicotine chewing gums, parches or other nicotine products

IN THE PAST, have you vaped, smoked or used nicotine products?

E-Cigarettes

Tobacco Cigarettes

Nicotine Inhalers, nicotine chewing gums, parches or other nicotine products

For peer review only

For peer review only

STROBE Statement—Checklist of items that should be included in reports of ***cross-sectional studies***

	Item No	Recommendation	Page No
**Title and abstract**	1	(*a*) Indicate the study's design with a commonly used term in the title or the abstract	1
		(*b*) Provide in the abstract an informative and balanced summary of what was done and what was found	2
**Introduction**			
Background/rationale	2	Explain the scientific background and rationale for the investigation being reported	4-6
Objectives	3	State specific objectives, including any prespecified hypotheses	6
**Methods**			
Study design	4	Present key elements of study design early in the paper	5
Setting	5	Describe the setting, locations, and relevant dates, including periods of recruitment, exposure, follow-up, and data collection	7
Participants	6	(*a*) Give the eligibility criteria, and the sources and methods of selection of participants	7
Variables	7	Clearly define all outcomes, exposures, predictors, potential confounders, and effect modifiers. Give diagnostic criteria, if applicable	7
Data sources/ measurement	8*	For each variable of interest, give sources of data and details of methods of assessment (measurement). Describe comparability of assessment methods if there is more than one group	*8*
Bias	9	Describe any efforts to address potential sources of bias	8
Study size	10	Explain how the study size was arrived at	8
Quantitative variables	11	Explain how quantitative variables were handled in the analyses. If applicable, describe which groupings were chosen and why	8
Statistical methods	12	(*a*) Describe all statistical methods, including those used to control for confounding	10
		(*b*) Describe any methods used to examine subgroups and interactions	N/A
		(*c*) Explain how missing data were addressed	N/A
		(*d*) If applicable, describe analytical methods taking account of sampling strategy	
		(*e*) Describe any sensitivity analyses	
**Results**			
Participants	13*	(a) Report numbers of individuals at each stage of study—eg numbers potentially eligible, examined for eligibility, confirmed eligible, included in the study, completing follow-up, and analysed	7
		(b) Give reasons for non-participation at each stage	7
		(c) Consider use of a flow diagram	
Descriptive data	14*	(a) Give characteristics of study participants (eg demographic, clinical, social) and information on exposures and potential confounders	7
		(b) Indicate number of participants with missing data for each variable of interest	7
Outcome data	15*	Report numbers of outcome events or summary measures	10
Main results	16	(*a*) Give unadjusted estimates and, if applicable, confounder-adjusted estimates and their precision (eg, 95% confidence interval). Make clear which confounders were adjusted for and why they were included	

For peer review only

		(*b*) Report category boundaries when continuous variables were categorized	
		(*c*) If relevant, consider translating estimates of relative risk into absolute risk for a meaningful time period	
Other analyses	17	Report other analyses done—eg analyses of subgroups and interactions, and sensitivity analyses	
**Discussion**			
Key results	18	Summarise key results with reference to study objectives	12-13
Limitations	19	Discuss limitations of the study, taking into account sources of potential bias or imprecision. Discuss both direction and magnitude of any potential bias	14-15
Interpretation	20	Give a cautious overall interpretation of results considering objectives, limitations, multiplicity of analyses, results from similar studies, and other relevant evidence	16
Generalisability	21	Discuss the generalisability (external validity) of the study results	
**Other information**			
Funding	22	Give the source of funding and the role of the funders for the present study and, if applicable, for the original study on which the present article is based	9

*Give information separately for exposed and unexposed groups.

Note: An Explanation and Elaboration article discusses each checklist item and gives methodological background and published examples of transparent reporting. The STROBE checklist is best used in conjunction with this article (freely available on the Web sites of PLoS Medicine at http://www.plosmedicine.org/, Annals of Internal Medicine at http://www.annals.org/, and Epidemiology at http://www.epidem.com/). Information on the STROBE Initiative is available at www.strobe-statement.org.

For peer review only

For peer review only - http://bmjopen.bmj.com/site/about/guidelines.xhtml

BMJ Open

A survey of the effect of viewing an on-line e-cigarette advertisement on attitudes towards cigarette and e-cigarette use in adults located in the UK and USA: a cross-sectional study

Journal:	*BMJ Open*
Manuscript ID	bmjopen-2018-027525.R2
Article Type:	Research
Date Submitted by the Author:	20-May-2019
Complete List of Authors:	Booth, Paula; University of East London, Psychology<br>Albery, Ian; London South Bank University Department of Applied Sciences, Division of Psychology (School of Applied Sciences)<br>Cox, Sharon; London South Bank University Department of Applied Sciences, Division of Psychology (School of Applied Sciences)<br>Frings, Daniel; London South Bank University Department of Applied Sciences, Division of Psychology (School of Applied Sciences)
**Primary Subject Heading**:	Public health
Secondary Subject Heading:	Smoking and tobacco, Public health
Keywords:	attitude, advertisement, e-cigarettes, tobacco, dual-users, smokers

SCHOLARONE™
Manuscripts

A survey of the effect of viewing an on-line e-cigarette advertisement on attitudes towards cigarette and e-cigarette use in adults located in the UK and USA: a cross-sectional study

Paula Booth[1], Ian. P. Albery[2], Sharon Cox[2] & Daniel Frings[2]

[1](Paula Booth – corresponding author) Applied Health and Communities College, School of Psychology, Psychological Sciences Department, The University of East London, Stratford Campus, Water Lane

London, E15 4LZ. p.booth@uel.ac.uk

Email: p.booth@uel.ac.uk

Phone: +44 (0)20 8223 4719

[2]Division of Psychology, School of Applied Sciences, London South Bank University, 103 Borough Road, London, SE1 0AA

Key words: E-Cigarette; Tobacco; Attitudes; Advertisement; Dual-Users

Word Count : 3822

For peer review only

Objectives: This study explored the potential for e-cigarette advertisements to (i) enhance attitudes towards cigarettes and/or (ii) reduce barriers to e-cigarettes uptake. The study tested whether exposure to an on-line electronic cigarette advertisement changed attitudes towards cigarettes and e-cigarettes in smokers, non-smokers, e-cigarette users and dual-users (smokers who also use e-cigarettes).

Design: Cross-sectional study

Setting: Online survey

Participants: Adults (n=964) aged 18 to 65 years old (*M* = 36 years, *SD* = 11.6) from the UK and USA. Participants were grouped into current non-smokers, e-cigarette users, dual-users and smokers.

Interventions: Participants viewed one of 15 randomly assigned online e-cigarette advertisements.

Primary measures: Three single 7 point Likert scales measuring health, desirability, social acceptability were completed pre and post advertisement exposure.

Results: Post exposure all smoking groups showed a decrease or no change in how socially acceptable or desirable they rated cigarettes. Paradoxically, dual-users rated cigarettes as being significantly healthier after viewing the advertisement (p =.01) whilst all other smoking group ratings remained the same. There was an increase or no change in how all smoking groups perceived the healthiness and desirability of e-cigarettes

Conclusions: We observed no evidence that exposure to an e-cigarette advertisement renormalizes or encourages smoking in smokers, non-smokers or e-cigarette users. However, there is some indication that viewing an e-cigarette advertisement may increase duals-users' perceptions of the health of smoking.

For peer review only

Strengths and limitations of this study

- This study is among the first to examine the attitudinal processes underlying the effect of viewing an advertisement on interest in both smoking and vaping.
- The sample were derived of smokers, non-smokers, vapers and dual users rather than focusing on only one smoking type which is often a limitation of the extant literature in this area of research.
- The effects of viewing an advertisement on actual smoking behaviour was not assessed and should be considered in future work.
- Multiple analyses increased the risk of Type 1 errors.
- The effects of different modalities of advertising were not assessed.

For peer review only

INTRODUCTION

There is a large body of evidence to support the notion that advertising cigarettes encourages people to start or to continue to smoke.[1,2] With the intent of reducing tobacco use globally, Article 13 of the WHO Framework Convention for Tobacco Control calls for "a comprehensive ban on advertising, promotion and sponsorship of cigarettes"[3] (pg. 11). However, this treaty does not cover e-cigarettes. While there are restrictions on advertising e-cigarettes in the UK (Committee on Advertising Practice)[4] and in the USA (U.S. Food and Drug Regulations)[5] e-cigarettes are still being marketed elsewhere and there is concern that the advertisements portray e-cigarettes as being glamourous.[6] In these territories the public now experiences increased exposure to e-cigarette advertisements[4,7,8] in the form of television, magazines, newspapers, billboards and internet advertisements.[9] The current paper explores the potential of such advertisements to (i) enhance attitudes towards cigarettes and (ii) reduce barriers to e-cigarette uptake amongst current smokers.

One concern related to e-cigarette advertisements is that they may renormalize smoking. The de-normalisation of smoking has been a cornerstone of tobacco control policies in encouraging existing smokers to quit and also to discourage initial uptake[10–12] and social acceptance of smoking has been shown to be a barrier to reduction in smoking prevalence in a variety of communities.[13,14] It is therefore important to determine

For peer review only

whether e-cigarette advertising may also affect attitudes towards cigarettes by making them more positive. A number of researchers have suggested that e-cigarette advertising could lead to increases in how socially acceptable and desirable cigarettes are perceived to be, and subsequently influence their continued use or possible (re)uptake in smokers, e-cigarette users and dual-users (those who use both cigarettes and e-cigarettes).[6,15–17]. Alongside observed effects on these groups, concerns that e-cigarette use may be a gateway to smoking suggests it is also important to determine whether advertising may encourage non-smokers to use e-cigarettes.[16,17] In this context, it is important to note that the evidence base around actual usage statistics currently suggests that e-cigarette availability does not appear to have led to regular use of either e-cigarettes or smoking amongst British non-smokers: the current number of UK non-smokers using an e-cigarette daily is less than 1%, and smoking prevalence rates continue to decline.[18] A similar pattern is observed in the US.[19] However, results from laboratory studies exploring the effects of advertisements on urges to smoke and interest in smoking amongst other groups are inconsistent, and none have examined attitudinal processes which may underlie those effects which have been observed.[20–22] The current study aims to examine the issue of renormalisation by measuring attitude change towards cigarettes in adult non-smokers, smokers, e-cigarette users and dual-users before and after viewing an advertisement.

For peer review only

In contrast to concerns about renormalisation, e-cigarette advertisements may represent an opportunity to promote reduced risk products as an alternative to smoking. Although there are currently only relatively few studies comparing the health effects of e-cigarettes use to smoking or non-smoking over time,[23,24] in the UK e-cigarettes are widely supported as a reduced risk nicotine alternative for smokers, and has received support from a number of agencies. [18,25–27] However in the UK, despite increasing public agency support, one barrier to the uptake of e-cigarettes amongst smokers has been an increasingly negative attitudes towards the products. Between 2015 and 2017 the proportion of UK smokers who believed that e-cigarettes are less harmful than cigarettes reduced from 31% to 20% (Action on Smoking and Health)[27]. Similarly, in the USA the percentage of smokers who perceived e-cigarettes to be equally harmful than combustible cigarettes increased from 23% in 2012 to 35% in 2015.[28] To the extent attitudes predict behaviour, such negative perceptions of the health of e-cigarettes are likely to reduce the potential for e-cigarettes to be used as a smoking cessation aid. E-cigarette advertising may serve to halt or reverse this decline. Research examining the effects of advertising on perception of e-cigarettes (carried out predominantly on younger populations) shows that viewing an advertisement may increase the perception of social acceptability and the intention to try an e-cigarette in the future.[7,29] For instance, 13-17 year old smokers showed a more positive attitude towards e-cigarettes after viewing an e-cigarette advertisement,[29] and adult smokers reported a decrease in

For peer review only

the acute urge to smoke[30] and an interest in trying e-cigarettes.[31–33] Thus, in the current study we also explore the potential for e-cigarette advertisements to change the way smokers (and other groups) perceive the healthiness, socially acceptability and general desirability of e-cigarettes.[13,34]

One limitation of the extant literature in these areas is that it focuses on either smokers, non-smokers and/or e-cigarette users. This leaves one important population, dual-users, understudied. In a survey of e-cigarette use in Europe, of an estimated 37 million e-cigarette users, 73% were currently still smoking.[35] Many dual-users use e-cigarettes in areas in which they are unable to smoke cigarettes.[36] However, they still have a preference for smoking cigarettes in stressful situations or for pleasure.[37] Thus, it is important to establish whether viewing an e-cigarette advertisement can change attitudes towards smoking in dual-users.

To explore the potential for-cigarette advertisements to (i) enhance attitudes towards cigarettes and/or (ii) reduce barriers to e-cigarettes uptake, the current study investigated whether viewing an e-cigarette advertisement influenced the extent to which smoking and e-cigarettes were perceived to be more or less healthy, desirable and socially acceptable in dual-users, smokers, non-smokers and e-cigarette users.

METHODS

Design

For peer review only

A cross sectional design in which attitude measures were taken pre and post advertisement viewing. The between-subjects factor was smoking group (non-smokers, e-cigarette users, dual-users and smokers). The dependent variables were the scores on a series of questions rating attitudes around the healthiness, desirability and social acceptability of both e-cigarettes and cigarettes.

Participants, Recruitment and Procedure

Originally, 964 participants completed the survey, between December 2015 and February 2016, but data from 199 were removed for being under the age of 18 or not entering their age, failing controls (see below) and having missing data. Data were included for 765 participants, 361 male, 400 female and 4 others from an age range of 18-65 years old (*M* = 36 years, *SD* = 11.6). Participants were defined as smokers (*n*=115), non-smokers (410), e-cigarette users (100) or dual-users (145). They were located in the USA (*n*=543) or the UK (*n*=222).

Participants were recruited through an online crowd-sourcing tool (Crowdflower, similar to MTurk). Crowdflower users were given information about the survey on the Crowdflower site before being given the opportunity to proceed by giving online consent to participate in the study and then clicking on the link to be transferred to a survey (delivered using Qualtrics). Crowdflower has an estimated pool of over 10,000 workers worldwide but response rates were unable to be calculated due to the age (18 to

For peer review only

65 years old) and location (UK or USA only) restrictions which would reduce the number of possible participants available.[38] Participants were paid a fee of $1.00 to complete the survey. Control questions were built into the Qualtrics survey to exclude automated responses and participants who were not concentrating appropriately on the questions (for example, “For this item please indicate strongly agree”). Additional controls were set up on the survey so that participants were unable to enter the site from the same IP address more than once and that they had to take a minimum time of 90 seconds to complete the survey. Post-hoc analysis indicated that a minimum of 100 participants (the smallest smoking group) was sufficient to detect an effect size r =.15 or greater, (with Z = 2.2 or above).

Patient and public involvement.

Patients were not involved in the development or conduct of this study. There are plans to disseminate the results to practitioners by including the results as an aspect in London South Bank courses provision aimed at service delivery managers and counsellors. A summary of the findings will be offered to Cancer Research UK and other policy organisations and promoted on relevant staff social media sites.

Measures

Attitudes towards Cigarettes

For peer review only

Participants rated on three single measures how *healthy*, how *desirable* and how *socially acceptable* they found cigarettes and e- cigarettes on a 7-point Likert type scale ranging from 1 '*strongly disagree'* to 7 '*strongly agree*.' They were given two separate statements: "When thinking about tobacco/e- cigarettes please indicate whether you think they are……" The order in which e-cigarettes and tobacco cigarette statements were presented were counter-balanced to reduce question order bias.

Smoking Status

Participants were asked to indicate how often (never, occasionally, very often, always) they smoked, vaped or used nicotine products both in the present and the past. Participants were placed in smoking groups based on their 'present' smoking status. Those that answered 'never' to using tobacco or e-cigarettes were defined as non-smokers, those that answered 'occasionally', 'very often' or 'always' were defined as smokers, e-cigarette users or dual-users as appropriate.

Procedure

The study was given ethical approval by the London South Bank University Research Ethics Committee (UREC1564). The data reported here were used to identify which advertisement would be used in a larger study. The current study and the larger study were funded by Cancer Research UK (CRUK grant number C54622/A20485).

For peer review only

Participants were asked to rate how healthy, desirable and socially acceptable they found tobacco and e-cigarettes before and after viewing one of fifteen e-cigarette advertisements (see below). In addition, after completing the post-test attitude questions they were asked to rate the emotional attributes and the perceived effectiveness of the advertisement and to give demographic characteristics. Finally, they were given a debrief about the study, a warning about the addictive nature of nicotine and given links to government quit smoking websites.

Advertisement Selection and Preparation

Each participant viewed one of fifteen advertisements which were allocated randomly in Qualtrics. The fifteen advertisements were chosen from a pool of 200 different advertisements displayed online between 2013 and 2016. Ten different themes of advertising were identified which depicted e-cigarettes as being; a smoking cessation tool, healthier than (tobacco) cigarettes, aesthetically pleasing, celebrity endorsed, sporty, an alternative to cigarettes in places where cigarettes were restricted, as satisfying, cheaper, more fragrant and as cool as cigarettes.[6] Five researchers coded each advertisement as one of the ten themes and advertisements were chosen that were consistently coded as the same theme by three of the five coders and which the research team found to be the most engaging. The final 15 advertisements included all ten themes, a variety of brands and images[31] and eight included a smoking or vaping cue and seven did not.[20]

For peer review only

RESULTS

Observation of Table 1 show that only 2.9% of non-smokers had been full-time smokers in the past and none had been full-time vapers. Only 10% of current e-cigarette users had not smoked in the past; most were either full-time or intermittent smokers previously. There were no smokers who had transferred from daily e-cigarette use to smoking, although 27.8% had used e-cigarettes intermittently in the past. Most dual-users had smoked or used e-cigarettes intermittently in the past.

Table 1. Percentage of previous tobacco and e-cigarette use of current non-smokers, e-cigarette users, smokers and dual-users.

Previous Use	*Status*	Current Non-smokers	Current E-cigarette User	Current Smoker	Current Dual-User
Tobacco Use	Never	68.5%	10.0%	0.9%	0.7%
	Intermittent	28.5%	49%	67.8%	72.9%
	Always	2.9%	41%	31.3%	26.4%
E-cigarette Use	Never	93.9%	21%	72.2%	5.7%
	Intermittent	6.1%	50%	27.8%	88.7%
	Always	0.0%	29%	0.0%	3.6%

Note: The categories 'occasionally' and 'very often' were collapsed and named intermittent

Health scores in the smokers group were not normally distributed due to the positive skew (baseline skewness = 2.63, test skewness = 2.40) thus Wilcoxon Signed Rank Tests were used to compare pre and post intervention scores in each smoking group (smokers, non-smokers, dual-users and e-cigarette users) for each outcome measure (health, desirability and social acceptability) of cigarettes and e-cigarettes(see Table 2).

Table 2. Mean healthy, desirability and social acceptability ratings (standard deviation in parentheses) towards tobacco and e-cigarettes by smoking group pre and post advertisement presentation.

		Sample							
Attitude target	*Attitude dimension*	Non-smoker (n=410)		E-cigarette user (n=100)		Smoker (n=115)		Dual-User (n= 145)	
		Pre	Post	Pre	Post	Pre	Post	Pre	Post
Cigarette	Healthy	1.27 (0.83)	1.28 (0.76)	1.47 (0.90)	1.47 (0.81)	1.59 (0.90)	1.73 (1.03)	2.08 (1.35)	**2.26**** **(1.50)**
	Desirability	1.86 (1.45)	1.77 (1.35)	2.84 (1.79)	**2.58*** **(1.67)**	3.52 (1.78)	**3.21**** **(1.67)**	3.85 (1.87)	3.71 (1.82)
	Social Acceptability	2.63 (1.57)	**2.39***** **(1.54)**	3.04 (1.76)	**2.86*** **(1.78)**	3.43 (1.55)	3.30 (1.64)	3.36 (1.67)	3.39 (1.77)
E-Cigarette	Healthy	2.53 (1.38)	**2.68**** **(1.51)**	4.79 (1.60)	4.82 (1.65)	3.03 (1.48)	**3.21***** **(1.54)**	3.80 (1.33)	**4.01**** **(1.42)**
	Desirability	2.76 (1.57)	**2.91*** **(1.70)**	5.36 (1.44)	5.21 (1.37)	3.91 (1.45)	3.91 (1.54)	4.82 (1.21)	**5.03**** **(1.24)**
	Social Acceptability	3.93 (1.58)	**3.81*** **(1.07)**	5.08 (1.45)	5.07 (1.46)	4.59 (1.39)	4.50 (1.40)	5.28 (1.05)	5.14 (1.18)

For peer review only

Note: Comparisons are made pre-post for each dimension for each group, with significant differences marked with asterisk(es): * = $p<0.05$ **= $p<0.005$ ***= $p<0.001$

Attitudes Towards Cigarettes

All smoking groups gave low scores for health of cigarettes at baseline. Showing an e-cigarette advertisement made no significant difference to the score in the smoking, non-smoking and e-cigarette user groups but dual-users scored cigarettes as healthier after viewing the advertisement ($Z = 2.57$ $p =.01$). There was no change in dual-users rating of desirability or social acceptability after viewing an advertisement. E-cigarette users scored cigarettes as significantly less desirable ($Z = -2.50$, $p =.013$) and less socially acceptable ($Z = -2.501$ $p =.012$) after viewing an advertisement. Smokers scored cigarettes as less desirable ($Z = -2.81$, $p =.005$) after viewing an advertisement but there was no difference in pre and post scores of social acceptability. Non-smokers showed no change in desirability scores of cigarettes but scored them as less socially acceptable ($Z = -4.67$ $p >.001$) after viewing an advertisement.

Non-smokers scored e-cigarettes as being healthier after viewing an e-cigarette advertisement ($Z = 2.97$, $p =.003$), more desirable ($Z = 2.60$, $p =.009$) but less socially acceptable ($Z = -2.12$ $p =.034$). E-cigarette users showed no change in attitudes. Smokers scored e-cigarettes as healthier after viewing the advertisements ($Z = 2.21$, p

For peer review only

=.027) but there was no change in desirability or social acceptability. Dual-users scored e-cigarettes as being healthier after viewing advertisement ($Z = 2.53$, p =.011) and more desirable ($Z = 2.04$, p =.042) but there was no change in social acceptability.

DISCUSSION

The current study aimed to explore the potential of e-cigarette advertising to (i) enhance attitudes towards cigarettes by reducing negative evaluations towards them and (ii) reduce barriers to the uptake of e-cigarettes by smokers. In terms of renormalisation by changes in attitude, little evidence in the current study supports the notion that viewing e-cigarette advertisements increased positive evaluations of cigarettes. Rather, perceptions of cigarettes being socially acceptable and desirable generally decreased. All smoking groups, other than dual-users, showed no change in health scores for cigarettes. However, these groups gave scores close to the scale's floor for health at baseline and there may have been no possibility to reduce scores further after viewing the advertisement. Thus, there was no indication that viewing an e-cigarette advertisement renormalized or encouraged smoking amongst these smokers, non-smokers and e-cigarette users.

After viewing an advertisement smokers scored cigarettes as less desirable and e-cigarettes as healthier supporting the notion that advertisements may reduce barriers to the uptake of e-cigarettes by smokers. Indeed, all groups scored e-cigarettes as healthier

For peer review only

after viewing the advertisement, other than e-cigarette users, whose baseline scores were almost at ceiling already. These results show that e-cigarette advertising successfully increases positive attitudes towards e-cigarettes. As perceptions of health are a key determinant in behaviour change these results encourage the view that smokers may potentially use e-cigarettes as a tool for smoking cessation.[39]

In non-smokers scores for social acceptability of both cigarettes and e-cigarettes decreased. This result is inconsistent with previous findings which found that when young people viewed e-cigarette advertisements the ratings for social acceptability increased.[7,29] This discrepancy in results may be due to the different age groups being tested and the suggestion that e-cigarette adverts (and products) are often designed with a younger target audience in mind.[40] It also mirrors a more general negative attitude towards e-cigarettes observed in the UK population in recent years.[27] However, in the current study, after viewing the advertisement the perception that e-cigarettes are healthy and desirable increased amongst non-smokers. Although widespread use of e-cigarettes by non-smokers has not been observed to date (in populations where data is available), this result suggests the effects of e-cigarettes advertising on actual e-cigarettes uptake amongst nicotine naïve users should continue to be monitored, especially in territories where advertising is prevalent.

E-cigarette users showed no change in attitudes towards e-cigarettes after viewing an advertisement. Perceptions of desirability and social acceptability of cigarettes

For peer review only

decreased after watching the advertisement. A large proportion of e-cigarette users had previously been smokers so these findings support the view that viewing an e-cigarette advertisement may help deter ex-smokers, who are vaping as a smoking cessation tool, from relapsing.

One group who are relatively under-researched (dual-users) displayed a different pattern of responses to cigarettes to the other smoking categories. They rated cigarettes and e-cigarettes as healthier after viewing the advertisement. The contradictory effect of the advertisements on the attitudes of this particular group suggests that the drive to use both tobacco and e-cigarettes is complex. Indeed, some research suggests that many dual-users may still use cigarettes because they find it to be a pleasurable experience and use e-cigarettes as a practical solution when smoking cigarettes is banned.[37] Although our initial explorations of this group warrant caution in their interpretation, they do flag the need for further research exploring how dual-users perceive and value the differences between e-cigarettes and smoking, and to identify the best strategy to help motivate this group to achieve cessation from combustible products.

Study Limitations

This was an exploratory study and we acknowledge that there were some limitations but that the current work can still contribute to advances in knowledge in the field. As the general public are likely to view a wide range of e-cigarette advertisement types, themes

For peer review only

and categories online we purposely chose to measure changes in attitudes to a variety of advertisements across broad smoking categories rather than assessing the effects of different types of e-cigarette advertisement, for example cue versus no cue, on specific smoking status. Future research could assess the effects of different types of e-cigarette advertisement on specific smoking status, for example daily versus intermittent smokers and vapers.

Due to the non-parametric nature of the data multiple analyses were carried out and thus there was an increased risk of Type 1 errors. After deliberation we chose not to use a statistical method to correct the experiment-wise error rate as this correction may then have increased the risk for Type 2 errors. As the purpose of this study was to explore the data with a view to providing a baseline from which to replicate the findings and inform further studies we considered that making a correction may hinder the accumulation of knowledge in this area and that increased Type 1 errors were preferential to increased Type 2 errors.[41,42]

In this study we measured attitudes towards cigarettes and e-cigarettes but did not assess any effects on intention to smoke or actual smoking behaviour. Recent evidence shows that viewing an e-cigarette product[43] or advertisement[44] may act as a cue that leads to an increased urge and desire to smoke or vape. Therefore, future research needs to investigate the relationship between attitude and cue reactivity. The current study had a cross-sectional design and the change in attitude was measured immediately after one

For peer review only

exposure to the advertisement so it cannot be determined whether effects of viewing an advertisement would persist over a longer time period. Future research would benefit from assessing changes in attitude longitudinally as well as measuring intentions to smoke or use e-cigarettes and actual smoking behaviour for a fixed period before and after viewing the advertisement.

Our outcomes used single measure attitude scales and although these had high external validity (and single item scales can be regarded as appropriate when constructs are well defined),[45] multiple items scales would have allowed empirical assessment of validity. Furthermore, measuring attitudes towards health using a seven-point Likert type scale resulted in possible floor effects. A more sensitive measure such as a Visual Analogue Scale may have given a broader range of data ensuring that the data was more normally distributed and giving a better indication of changes that may have occurred at the lower end of the scale.

There is a social stigma associated with smoking and so there may have been an effect of social desirability bias. Attitudes in the current study were assessed using explicit, self-report measures so an implicit measure of attitude could be measured in future research as previous research shows that these measures of attitude interact to predict actual smoking behaviour.[46] Additionally, it was considered whether results may be a consequence of the boomerang effect. The advertisements trying to persuade e-cigarettes to be viewed more positively may have inadvertently caused the consumer to

For peer review only

resist the persuasion attempt and instead view e-cigarettes more negatively or cigarettes more positively, as a form of non-compliance. Results were not unexpected, other than for dual-users, and so the use of a control group in future research may help to elucidate more of an understanding of attitude changes in response to advertisements in this particular group.

Conclusion

The results from this study suggest that viewing an e-cigarette advertisement is unlikely to renormalize smoking amongst most groups. However, we observed some evidence that e-cigarette advertisements may increase how healthy cigarettes are perceived amongst dual-users. This highlights the importance of more research with this relatively understudied group, in particular around factors which promote the decision to transition from dual-use to e-cigarette use or nicotine abstinence. In terms of reducing barriers to uptake, e-cigarette advertisement may encourage smokers to quit using e-cigarettes as a smoking cessation tool. Thus, e-cigarette advertisements could be considered a viable tool to stimulate or support smoking cessation. However, where e-cigarette advertising is prevalent, careful monitoring of uptake of e-cigarettes amongst nicotine naïve populations is important.

References

For peer review only

1 Evans N, Farkas A, Gilpin E, *et al.* Influence of Tobacco Marketing and Exposure to Smokers on Adolescent Susceptibility to Smoking. *JNCI J Natl Cancer Inst* 1995;**87**:1538–45. doi:10.1093/jnci/87.20.1538

2 Tye JB, Warner KE, Glantz SA. Tobacco Advertising and Consumption: Evidence of a Causal Relationship. *J Public Health Policy* 1987;**8**:492. doi:10.2307/3342275

3 WHO FRAMEWORK CONVENTION ON TOBACCO CONTROL WHO Library Cataloguing-in-Publication Data. Published Online First: 2010.http://apps.who.int/iris/bitstream/handle/10665/42811/9241591013.pdf;jsessionid=62CFB43C3F37022BD16CEA88E7D3DF93?sequence=1 (accessed 16 Apr2018).

4 New rules for the marketing of e-cigarettes CAP and BCAP's Joint Regulatory Statement. https://www.asa.org.uk/asset/851604F6-C081-420C-9DDDA65124B775A4/ (accessed 21 Aug2017).

5 Commissioner O of the. Economic Impact Analyses of FDA Regulations - Summary: Deeming Tobacco Products to be Subject to the Food, Drug, and Cosmetic Act, as Amended by the Family Smoking Prevention and Tobacco Control Act; Regulations Restricting the Sale and Distribution of Tobacco Products and Required Warning Statements for Tobacco Product Packages and Advertisements (Final Rule). https://www.fda.gov/AboutFDA/ReportsManualsForms/Reports/EconomicAnalyses/ucm500249.htm (accessed 16 Apr2018).

6 De Andrade M, Hastings G, Angus K, *et al.* the marketing of electronic cigarettes in the uk. Published Online First: 2013.https://dspace.stir.ac.uk/bitstream/1893/17889/1/deAndradeetale-cigsreport.pdf (accessed 26 Mar2017).

7 Duke JC, Lee YO, Kim AE, *et al.* Exposure to Electronic Cigarette Television Advertisements Among Youth and Young Adults. *Pediatrics* 2014;**134**.http://pediatrics.aappublications.org/content/134/1/e29.full (accessed 21 Aug2017).

8 Basch CH, Mongiovi J, Hillyer GC, *et al.* An Analysis of Electronic Cigarette and Cigarette Advertising in US Women's Magazines. *Int J Prev Med* 2016;**7**:103. doi:10.4103/2008-7802.190089

9 Bauld L, Angus K, De Andrade M, *et al.* Electronic Cigarette Marketing: Current Research and Policy. 2016. http://www.cancerresearchuk.org/sites/default/files/electronic_cigarette_marketing_report_final.pdf (accessed 21 Aug2017).

10 Linkenbach, J. W., & Perkins HW. MOST of us are tobacco free: An eight-month social norms campaign reducing youth initiation of smoking in Montana.

In: H. W. Perkins, ed. *The social norms approach to preventing school and college age substance abuse: A handbook for educators, counselors, and clinicians*. San Francisco, CA, US: : Jossey-Bass 2003. 224–34.http://psycnet.apa.org/record/2003-02237-013 (accessed 16 Apr2018).
11 Reid JL, Manske SR, Leatherdale ST. Factors related to adolescents' estimation of peer smoking prevalence. *Health Educ Res* 2007;**23**:81–93. doi:10.1093/her/cym006
12 Bayer R, Bachynski KE. Banning Smoking In Parks And On Beaches: Science, Policy, And The Politics Of Denormalization. *Health Aff* 2013;**32**:1291–8. doi:10.1377/hlthaff.2012.1022
13 Nicholson AK, Borland R, van der Sterren AE, *et al.* Social acceptability and desirability of smoking in a national sample of Aboriginal and Torres Strait Islander people. *Med J Aust* 2015;**202**:57–62. doi:10.5694/mja14.01534
14 Twyman L, Bonevski B, Paul C, *et al.* Perceived barriers to smoking cessation in selected vulnerable groups: a systematic review of the qualitative and quantitative literature. *BMJ Open* 2014;**4**:e006414. doi:10.1136/bmjopen-2014-006414
15 Fairchild AL, Bayer R, Colgrove J. The Renormalization of Smoking? E-Cigarettes and the Tobacco "Endgame". *N Engl J Med* 2014;**370**:293–5. doi:10.1056/NEJMp1313940
16 Schneider S, Diehl K. Vaping as a Catalyst for Smoking? An Initial Model on the Initiation of Electronic Cigarette Use and the Transition to Tobacco Smoking Among Adolescents. *Nicotine Tob Res* 2016;**18**:647–53. doi:10.1093/ntr/ntv193
17 Etter J-F. Gateway effects and electronic cigarettes. *Addiction* Published Online First: 7 August 2017. doi:10.1111/add.13924
18 Mcneill A, Brose LS, Calder R, *et al.* Evidence review of e-cigarettes and heated tobacco products 2018 A report commissioned by Public Health England. Published Online First: 2018.https://assets.publishing.service.gov.uk/government/uploads/system/uploads/attachment_data/file/684963/Evidence_review_of_e-cigarettes_and_heated_tobacco_products_2018.pdf (accessed 16 Apr2018).
19 *QuickStats:* Cigarette Smoking Status* Among Current Adult E-cigarette Users,† by Age Group — National Health Interview Survey,§ United States, 2015. *MMWR Morb Mortal Wkly Rep* 2016;**65**:1177. doi:10.15585/mmwr.mm6542a7
20 Maloney EK, Cappella JN. Does Vaping in E-Cigarette Advertisements Affect Tobacco Smoking Urge, Intentions, and Perceptions in Daily, Intermittent, and Former Smokers? *Health Commun* 2015;:1–10.
21 Durkin SJ, Bayly M, Wakefield MA. Can E-cigarette Ads Undermine Former Smokers? An Experimental Study. *Tob Regul Sci* 2016;**2**:263–77. doi:10.18001/TRS.2.3.6
22 Smith DM, Bansal-Travers M, O'Connor RJ, *et al.* Associations between

For peer review only

perceptions of e-cigarette advertising and interest in product trial amongst US adult smokers and non-smokers: results from an internet-based pilot survey. *Tob Induc Dis* 2015;**13**:14. doi:10.1186/s12971-015-0039-6
23 Shahab L, Goniewicz ML, Blount BC, *et al.* Nicotine, Carcinogen, and Toxin Exposure in Long-Term E-Cigarette and Nicotine Replacement Therapy Users. *Ann Intern Med* 2017;**166**:390. doi:10.7326/M16-1107
24 Polosa R, Cibella F, Caponnetto P, *et al.* Health impact of E-cigarettes: a prospective 3.5-year study of regular daily users who have never smoked. *Sci Rep* 2017;**7**:13825. doi:10.1038/s41598-017-14043-2
25 E-cigarettes | Cancer Research UK. http://www.cancerresearchuk.org/about-cancer/causes-of-cancer/smoking-and-cancer/e-cigarettes (accessed 16 Apr2018).
26 Royal College of Physicians. Nicotine without smoke: Tobacco harm reduction | RCP London. https://www.rcplondon.ac.uk/projects/outputs/nicotine-without-smoke-tobacco-harm-reduction-0/ (accessed 18 Dec2017).
27 ASH Action on Smoking and Health. Use of electronic cigarettes (vapourisers) among adults in Great Britain. ASH Fact Sheet 2017. 2017.
28 Majeed B, Weaver S, … KG-A journal of, *et al.* Changing perceptions of harm of e-cigarettes among US adults, 2012–2015. *Elsevier*https://scholar.google.co.uk/scholar?hl=en&as_sdt=0%2C5&q=Changing+perceptions+of+harm+of+e-cigarettes+among+US+adults%2C+2012–2015&btnG= (accessed 16 Apr2018).
29 Farrelly MC, Duke JC, Crankshaw EC, *et al.* A Randomized Trial of the Effect of E-cigarette TV Advertisements on Intentions to Use E-cigarettes. *Am J Prev Med* 2015;**49**:686–93. doi:10.1016/j.amepre.2015.05.010
30 Jo CL, Golden SD, Noar SM, *et al.* Effects of E-cigarette Advertising Messages and Cues on Cessation Outcomes. *Tob Regul Sci* 2018;**4**:562–72. doi:10.18001/TRS.4.1.3
31 Pepper JK, Brewer NT. Electronic nicotine delivery system (electronic cigarette) awareness, use, reactions and beliefs: a systematic review. *Tob Control* 2014;**23**:375–84. doi:10.1136/tobaccocontrol-2013-051122
32 Kim AE, Lee YO, Shafer P, *et al.* Adult smokers' receptivity to a television advert for electronic nicotine delivery systems. *Tob Control* 2015;**24**:132–5. doi:10.1136/tobaccocontrol-2013-051130
33 Smith DM, Bansal-Travers M, O'Connor RJ, *et al.* Associations between perceptions of e-cigarette advertising and interest in product trial amongst US adult smokers and non-smokers: results from an internet-based pilot survey. *Tob Induc Dis* 2015;**13**:14. doi:10.1186/s12971-015-0039-6
34 Chapman S, Freeman B. Markers of the denormalisation of smoking and the tobacco industry. *Tob Control* 2008;**17**:25–31. doi:10.1136/tc.2007.021386
35 Farsalinos KE, Poulas K, Voudris V, *et al.* Electronic cigarette use in the

For peer review only

European Union: analysis of a representative sample of 27 460 Europeans from 28 countries. *Addiction* 2016;**111**:2032–40. doi:10.1111/add.13506

36 Adriaens K, Van Gucht D, Baeyens F. Differences between Dual Users and Switchers Center around Vaping Behavior and Its Experiences Rather than Beliefs and Attitudes. *Int J Environ Res Public Health* 2017;**15**:12. doi:10.3390/ijerph15010012

37 Maglia M, Caponnetto P, Di Piazza J, *et al.* Dual use of electronic cigarettes and classic cigarettes: a systematic review. *Addict Res Theory* 2017;:1–9. doi:10.1080/16066359.2017.1388372

38 Peer E, Brandimarte L, Samat S, *et al.* Beyond the Turk: Alternative platforms for crowdsourcing behavioral research. *J Exp Soc Psychol* 2017;**70**:153–63. doi:10.1016/j.jesp.2017.01.006

39 Webb TL, Joseph J, Yardley L, *et al.* Using the internet to promote health behavior change: a systematic review and meta-analysis of the impact of theoretical basis, use of behavior change techniques, and mode of delivery on efficacy. *J Med Internet Res* 2010;**12**:e4. doi:10.2196/jmir.1376

40 Richardson A, Ganz O, Vallone D. Tobacco on the web: surveillance and characterisation of online tobacco and e-cigarette advertising. *Tob Control* 2015;**24**:341–7. doi:10.1136/tobaccocontrol-2013-051246

41 Armstrong RA. When to use the Bonferroni correction. *Ophthalmic Physiol Opt* 2014;**34**:502–8. doi:10.1111/opo.12131

42 Nakagawa S. A farewell to Bonferroni: the problems of low statistical power and publication bias. *Behav Ecol* 2004;**15**:1044–5. doi:10.1093/beheco/arh107

43 King AC, Smith LJ, McNamara PJ, *et al.* Second Generation Electronic Nicotine Delivery System Vape Pen Exposure Generalizes as a Smoking Cue. *Nicotine Tob Res* 2017;**20**:ntw327. doi:10.1093/ntr/ntw327

44 Chen Y, Fowler CH, Papa VB, *et al.* Adolescents' behavioral and neural responses to e-cigarette advertising. *Addict Biol* 2018;**23**:761–71. doi:10.1111/adb.12510

45 Postmes T, Haslam SA, Jans L. A single-item measure of social identification: Reliability, validity, and utility. *Br J Soc Psychol* 2013;**52**:597–617. doi:10.1111/bjso.12006

46 Perugini M. Predictive models of implicit and explicit attitudes. *Br J Soc Psychol* 2005;**44**:29–45. doi:10.1348/014466604X23491

Funding statement: 'This work was supported by Cancer Research UK grant number C54622/A20485'.

For peer review only

Competing Interest Statement: Sharon Cox has provided consultancy work for the Pacific Life insurance group on smoking and reduced risk product use and prevalence rates. Daniel Frings and Ian Albery are both investigators on a randomised controlled trial funded by Allen Carrs Easyway Ltd. This trial is comparing the Allen Carr Easway stop-smoking method to local NHS 1-1 stop smoking counselling service (ISRCTN23584477). The team are contractually free to publish the results regardless of the study outcome. They have no other conflicts of interest to declare.

Authors' contributions: Dr Frings and Prof. Albery conceptualized and designed the study and critically reviewed and revised the manuscript; Dr Cox, critically reviewed and revised the manuscript; Dr Booth drafted the initial manuscript and administered the testing, data collection and statistical analysis. All authors approved the final manuscript as submitted.

Data sharing statement: Extra data is available by emailing p.booth@uel.ac.uk or fringsd@lsbu.ac.uk

For peer review only

STROBE Statement—Checklist of items that should be included in reports of ***cross-sectional studies***

	Item No	Recommendation	Page No
**Title and abstract**	1	(*a*) Indicate the study's design with a commonly used term in the title or the abstract	1
		(*b*) Provide in the abstract an informative and balanced summary of what was done and what was found	2
**Introduction**			
Background/rationale	2	Explain the scientific background and rationale for the investigation being reported	4-7
Objectives	3	State specific objectives, including any prespecified hypotheses	7
**Methods**			
Study design	4	Present key elements of study design early in the paper	5
Setting	5	Describe the setting, locations, and relevant dates, including periods of recruitment, exposure, follow-up, and data collection	8
Participants	6	(*a*) Give the eligibility criteria, and the sources and methods of selection of participants	8
Variables	7	Clearly define all outcomes, exposures, predictors, potential confounders, and effect modifiers. Give diagnostic criteria, if applicable	8
Data sources/ measurement	8*	For each variable of interest, give sources of data and details of methods of assessment (measurement). Describe comparability of assessment methods if there is more than one group	*10*
Bias	9	Describe any efforts to address potential sources of bias	10
Study size	10	Explain how the study size was arrived at	9
Quantitative variables	11	Explain how quantitative variables were handled in the analyses. If applicable, describe which groupings were chosen and why	8/13
Statistical methods	12	(*a*) Describe all statistical methods, including those used to control for confounding	13
		(*b*) Describe any methods used to examine subgroups and interactions	13
		(*c*) Explain how missing data were addressed	8
		(*d*) If applicable, describe analytical methods taking account of sampling strategy	Not Applicable
		(*e*) Describe any sensitivity analyses	Not Applicable
**Results**			
Participants	13*	(a) Report numbers of individuals at each stage of study—eg numbers potentially eligible, examined for eligibility, confirmed eligible, included in the study, completing follow-up, and analysed	8
		(b) Give reasons for non-participation at each stage	8
		(c) Consider use of a flow diagram	Not Applicable
Descriptive data	14*	(a) Give characteristics of study participants (eg demographic, clinical, social) and information on exposures and potential confounders	8
		(b) Indicate number of participants with missing data for each variable of interest	8

For peer review only - http://bmjopen.bmj.com/site/about/guidelines.xhtml

Outcome data	15*	Report numbers of outcome events or summary measures	13
Main results	16	(*a*) Give unadjusted estimates and, if applicable, confounder-adjusted estimates and their precision (eg, 95% confidence interval). Make clear which confounders were adjusted for and why they were included	Not Applicable
		(*b*) Report category boundaries when continuous variables were categorized	Not Applicable
		(*c*) If relevant, consider translating estimates of relative risk into absolute risk for a meaningful time period	Not Applicable
Other analyses	17	Report other analyses done—eg analyses of subgroups and interactions, and sensitivity analyses	Not Applicable
**Discussion**			
Key results	18	Summarise key results with reference to study objectives	14-15
Limitations	19	Discuss limitations of the study, taking into account sources of potential bias or imprecision. Discuss both direction and magnitude of any potential bias	17-18
Interpretation	20	Give a cautious overall interpretation of results considering objectives, limitations, multiplicity of analyses, results from similar studies, and other relevant evidence	20
Generalisability	21	Discuss the generalisability (external validity) of the study results	18-19
**Other information**			
Funding	22	Give the source of funding and the role of the funders for the present study and, if applicable, for the original study on which the present article is based	10

*Give information separately for exposed and unexposed groups.

Note: An Explanation and Elaboration article discusses each checklist item and gives methodological background and published examples of transparent reporting. The STROBE checklist is best used in conjunction with this article (freely available on the Web sites of PLoS Medicine at http://www.plosmedicine.org/, Annals of Internal Medicine at http://www.annals.org/, and Epidemiology at http://www.epidem.com/). Information on the STROBE Initiative is available at www.strobe-statement.org.

For peer review only

For peer review only - http://bmjopen.bmj.com/site/about/guidelines.xhtml
